# Transcriptomic and physiological responses of *Quercus acutissima* and *Quercus palustris* to drought stress and rewatering

**DOI:** 10.3389/fpls.2024.1430485

**Published:** 2024-08-06

**Authors:** Tae-Lim Kim, Changyoung Oh, Michael Immanuel Jesse Denison, Sathishkumar Natarajan, Kyungmi Lee, Hyemin Lim

**Affiliations:** ^1^ Department of Forest Bioresources, National Institute of Forest Science, Suwon, Republic of Korea; ^2^ Bigdata Design Group, 3BIGS Company Limited, Hwaseong, Republic of Korea

**Keywords:** *Quercus acutissima*, *Quercus palustris*, transcriptome, physiological response, drought stress, recovery, oak

## Abstract

Establishment of oak seedlings, which is an important factor in forest restoration, is affected by drought that hampers the survival, growth, and development of seedlings. Therefore, it is necessary to understand how seedlings respond to and recover from water-shortage stress. We subjected seedlings of two oak species, *Quercus acutissima* and *Quercus palustris*, to drought stress for one month and then rewatered them for six days to observe physiological and genetic expression changes. Phenotypically, the growth of *Q. acutissima* was reduced and severe wilting and recovery failure were observed in *Q. palustris* after an increase in plant temperature. The two species differed in several physiological parameters during drought stress and recovery. Although the photosynthesis-related indicators did not change in *Q. acutissima*, they were decreased in *Q. palustris*. Moreover, during drought, content of soluble sugars was significantly increased in both species, but it recovered to original levels only in *Q. acutissima*. Malondialdehyde content increased in both the species during drought, but it did not recover in *Q. palustris* after rewatering. Among the antioxidant enzymes, only superoxide dismutase activity increased in *Q. acutissima* during drought, whereas activities of ascorbate peroxidase, catalase, and glutathione reductase increased in *Q. palustris*. Abscisic acid levels were increased and then maintained in *Q. acutissima*, but recovered to previous levels after rewatering in *Q. palustris*. RNA samples from the control, drought, recovery day 1, and recovery day 6 treatment groups were compared using transcriptome analysis. *Q. acutissima* exhibited 832 and 1076 differentially expressed genes (DEGs) related to drought response and recovery, respectively, whereas *Q. palustris* exhibited 3947 and 1587 DEGs, respectively under these conditions. Gene ontology enrichment of DEGs revealed “response to water,” “apoplast,” and “Protein self-association” to be common to both the species. However, in the heatmap analysis of genes related to sucrose and starch synthesis, glycolysis, antioxidants, and hormones, the two species exhibited very different transcriptome responses. Nevertheless, the levels of most DEGs returned to their pre-drought levels after rewatering. These results provide a basic foundation for understanding the physiological and genetic expression responses of oak seedlings to drought stress and recovery.

## 1 Introduction

Plants encounter biotic and abiotic stress throughout their lives. They have evolved adaptive responses to cope with environmental stress, such as drought, salinity, and temperature fluctuations (He et al., 2018). With increasing prevalence and severity of drought associated with climate change, the frequency and intensity of drought events is predicted to increase globally (Naumann et al., 2018). Drought stress is the primary abiotic stress that can exacerbate other abiotic stresses and may occur concurrently with them (Cruz de Carvalho, 2008).

Plants exhibit varying degrees of drought tolerance, which differs among species. Various strategies have been devised to enhance the water adsorption and stress tolerance of plants. Severe drought can lead to plant dehydration, resulting in wilting, and ultimately, death (Fang and Xiong, 2015). Drought-induced water shortage is the most fatal abiotic stress that profoundly affects plant growth, development, reproductive capacity, and survival. Therefore, understanding drought stress response of plants and mechanisms governing stress signal perception and transduction is important in preparing for water shortages due to desertification. Water scarcity is recognized at the molecular and cellular levels in plant roots, and signals are transmitted to the shoots to respond to and prepare for stressful environments. Stress induces coordinated and interactive responses in plants, significantly affecting gene expression leading to the modulation of proteins and metabolites (Atkinson and Urwin, 2012). The selection and development of drought-tolerant plants are coveted goals of researchers engaged in generating drought-tolerant plants.

The effects of drought stress on plant adaptations have been extensively investigated and changes in crop yield, growth, pigment synthesis, photosynthetic activity, membrane integrity, osmotic adjustment, stomatal regulation, cell division, and reactive oxygen species (ROS) accumulation have been highlighted (Shinozaki and Yamaguchi-Shinozaki, 2007). Water-use efficiency is compromised and CO_2_ assimilation by leaves is hindered by factors such as stomatal closure, membrane damage, and altered enzyme functioning (Flexas and Medrano, 2002). Enhanced flux through the photorespiratory pathway during stress generates ROS that oxidatively damage nucleic acids, proteins, and lipids (Xiong and Zhu, 2002). Environmental conditions during the seedling stage strongly influence the survival and growth of plants throughout their lifespan (Danby and Hik, 2007). Because trees have long growth periods, selecting drought-tolerant varieties at the seedling stage is of utmost importance with regard to cost and time. Therefore, researchers have focused on drought-induced physiological and metabolic responses in seedlings, and have explored screening factors for drought resistance. The maintenance and expansion of forests is very important for the prevention and mitigation of desertification. Increasing the survival rate of seedlings is the most important factor in afforestation. Therefore, further understanding of the seedling response to drought is required.

Various responses, including reduced growth rate, increased ROS levels, changes in net photosynthetic rate, decrease in Fv/Fm, photosynthetic function, and abscisic acid (ABA) content, and stomatal pore narrowing, are induced during drought stress. The drought-induced changes are recovered upon rewatering (Izanloo et al., 2008; Xu et al., 2009). However, the degree of recovery and time required for it vary depending on the severity and duration of drought (Xu et al., 2010). The presence of a pre-drought limitation (PDL) has been suggested, and studies have indicated that plants experiencing prolonged or severe drought may not fully recover their final biomass or leaf area compared with that of controls (Xu et al., 2009). The recovery upon rewatering might be influenced by the intensity or duration of pre-drought conditions. Moreover, the limitations observed after rewatering can be attributed to constraints in the meristem of plant tissues (Yahdjian and Sala, 2006). Several studies have confirmed the phenomenon of overcompensation in plant growth following rewatering after drought (Siopongco et al., 2006; Xu and Zhou, 2007). However, research on plant recovery after drought has several unknown aspects. *Quercus acutissima* Carruth. (sawtooth oak) and *Quercus plastris* Münchh. (pin oak) are dominant oak species widely distributed across East Asian countries, including Korea, China, and Japan (Jang et al., 2023; Yuan et al., 2023). These species have significant economic and ecological value in warm-temperate deciduous broad-leaved woodlands (Zhang et al., 2015). They have been proposed as indicator species for assessing the health of local forests, highlighting their ecological significance and impact on the local ecology (Ma et al., 2022). In Korea, both species are major deciduous timber species and their acorns are used as food resources for humans and wildlife (Hong et al., 2010; Noh et al., 2020). *Q. acutissima* is a large deciduous tree found in hilly regions spanning from South Korea to Japan. It commonly grows in various plant communities and often forms pure stands. It is a pioneer species that takes advantage of the gaps created by disturbances during colonization (Yamamoto et al., 2010). It contributes to charcoal production and is a valuable source of architectural materials (Cha et al., 2017). *Q. plastris* inhabits Korea and the central and eastern parts of the United States, predominantly the floodplains (Black, 1984). It is a lowland oak species, which grows well in a variety of moist, poorly drained soils (Kusi and Karsai, 2020). *Q. plastris* wood is used in building materials and as firewood. Considering its usefulness, it has been explored for effective afforestation and survival (Hayford et al., 2023). Recently, genetic information on *Q. palustris* was reported (Barta et al., 2017); however, genetic and biochemical studies remain scarce.

In a recent study, *Quercus lobata* Née, grown in different regions, was reported to exhibit differential gene expression depending on its region of origin in response to drought (Gugger et al., 2017). Analysis of root tissue of *Quercus suber* exposed to drought revealed induction of the core ABA signaling pathway involving *PP2C-SnRK2-ABF* components as a mechanism of drought tolerance (Magalhães et al., 2016). Two reference genes (At1g54610 in three species, *TOPP2* in *Quercus ilex*, *U2AF35B* in *Quercus pubescens*, and *FHY3* in *Quercus robur*) were selected from leaves of three European oak species exposed to drought stress (Kotrade et al., 2019). In drought-stressed *Q. ilex* seedlings, the expression of *FtSH6*, *CLPB1*, *CLPB3*, and HSP22 was increased at both transcript and protein levels, suggesting their relevance as markers (Guerrero-Sánchez et al., 2021).

Among the oak species, we selected *Q. acutissima*, which is widely distributed in dry areas, and *Q. plastris*, which is distributed in relatively humid areas. The experiment was planned with the expectation that the differences in distribution of these two tree species would result in differences in drought tolerance and recovery within oak trees. In the present study, we examined the response of oak seedlings to prolonged drought stress and their recovery upon rewatering. Phenotypic and physiological changes were evaluated. In addition, changes in the content of typical drought stress indicators and antioxidants in plants were analyzed. Transcriptome analysis of the genes related to drought stress and recovery was performed. We report comprehensive findings on how oaks respond phenotypically, genetically, and physiologically to drought stress and how they recover to some extent upon rewatering.

## 2 Materials and methods

### 2.1 Plant materials and growth conditions

The experiment was conducted for two months at the National Institute of Forest Science in Suwon, Korea (37°15′04″N, 136°57′59″E) under semicontrolled conditions. Seeds were sown in the open field in April 2020 and replanted in March 2021. In 2022, after transplanting into pots, the experiment was started after two months of acclimatization. Oak seedlings were grown in a greenhouse on top-soil and sand (3:1) mixture in pots (perlite and vermiculite (2:1:1), 11 cm × 11 cm × 3.14 × 24 cm) with appropriate soil moisture. The temperature of the greenhouse was maintained at 22–29°C. Three replicates were performed for each experiment. For each replicate, leaves of 12 plants (control: 3 replicates × 12 plants; drought: 3 replicates × 12 plants) were pooled and analyzed. Two months after 1-year-old plantlets were transferred to pots, healthy and uniform plantlets were randomly selected from each treatment group. Plants were watered to achieve 40% soil moisture one day before the drought treatment. The control group was given sufficient water once every two or three days, and the drought treatment group was provided with good drainage but was not irrigated for 31 days. The plants were then deprived of water for 31 d and rewatered for 6 d. At the end of drought treatment, the soil moisture content, measured using a moisture probe (ICT International Pty., Ltd., Armidale, NSW, Australia), decreased to less than 3% and was maintained at approximately 20–30% after rewatering. Leaves were harvested simultaneously from May to July 2022, around 9:00 am to 11:00 am. The upper and lower leaves were harvested and mixed at the same stage, and the same samples were used in all experiments. Samples were stored at −80°C in a cryotic refrigerator until the experiment.

### 2.2 Thermal imaging and relative water content of leaves

Leaf temperature was measured using infrared (IR) digital imaging with a Fluke TiX560 thermal imaging camera (Fluke Corp., Everett, WA, USA) on the 31st day of the drought treatment and on the 1st and 6th day of rewatering. RWC (%) represents the ratio of the water content in the normal growth state of the leaf to the maximum water content. The fresh weight (FW) of the leaves was measured and then they were kept immersed in a Petri dish containing distilled water for 24 h. Water on the leaf surface was removed, and the saturated weight (TW) was measured. After drying in a dryer at 70°C for 24 h, the dry weight (DW) of the leaves was measured, and the RWC was calculated using the formula given below (Turner, 1986).


RWC (%)=(FW−DW)/(TW−DW)×100

### 2.3 Measurement of chlorophyll fluorescence and content

The maximum efficiency of photosystem II (PSII) chemistry, indicated by Fv/Fm, and the potential activity of PSII were assessed using the Kautsky induction method with a portable Handy FluorCam instrument (Photon System Instruments Ltd., Brno, Czech Republic) (Kautsky and Hirsch, 1931). Measurements were conducted once every 3–5 days on 12 plants at similar growth stages. To induce chlorophyll fluorescence, leaves were dark-adapted for 15 min, and then exposed to a 5-second pulse of irradiation (1,500 µmol•m^−2^•s^−1^). The fluorescence variables, Fo, Fm, Fv/Fo, and Fv/Fm, were recorded and analyzed. Photosynthetic pigment content was determined using a previously described method (Sibley et al., 1996). Fresh leaves (0.1 g) were collected in triplicate for each measurement. Supernatants obtained from leaf extracts were measured at 470, 647, and 664 nm using a Biospectrometer (Eppendorf, Hamburg, Germany). The concentrations of photosynthetic pigments were calculated using the following formula:


Chlorophyll a=12.7A664−2.79A647

Chlorophyll b=20.7A647−4.62A664

Carotenoids=(1000A470−1.82Chl a−85.02Chl b)/198

where A represents the absorbance, and pigment concentrations are reported as mg/g FW.

### 2.4 Extraction and measurement of soluble sugar

For extracting total soluble sugars, fresh leaf samples (0.1 g) were collected in triplicate and extracted in 80% ethanol using a modification of a previously described method (Irigoyen et al., 1992). The total soluble sugar content was measured at 620 nm using a Biospectrometer (Eppendorf), with glucose as a standard, and expressed as mg/g FW. For extracting glucose, fructose, and sucrose, 0.1 g fresh leaf samples were collected in triplicate and the sugars were extracted as described previously (Lu and Sharkey, 2004). Sugar concentrations were measured using a Biospectrometer (Eppendorf), as described previously (Stitt et al., 1989). For starch content, the sediments obtained from aqueous ethanol extractions were autoclaved for 3 h in distilled H_2_O. The resulting solution was enzymatically digested to glucose using α-amylase and amyloglucosidase in the Total Starch Kit (Megazyme International Ireland Ltd., Wicklow, Ireland; K-TSTA-100A) as described previously (Walters et al., 2004). Sugar concentrations were determined enzymatically (Stitt et al., 1989).

### 2.5 Measurement of proline, malondialdehyde, hydrogen peroxide, superoxide dismutase, catalase, ascorbate peroxidase, peroxidase, glutathione reductase, ABA, and indole-3-acetic acid

For proline extraction, fresh leaves (0.5 g) were homogenized in 3% (w/v) aqueous sulfosalicylic acid. Proline content was estimated using ninhydrin reagent following a previously described method (Bates et al., 1973). Absorbance of the reaction mixture was measured at 520 nm. Proline concentration was determined using a calibration curve and expressed as mmol proline g^-1^ FW. For measuring the MDA content, leaves were extracted with a mixture of 20% trichloroacetic acid (w/v) and 0.5% thiobarbituric acid (w/v). The extract was then heated at 95°C for 40 min, followed by cooling on ice, and centrifuged at 14,000 × *g* for 10 min. The absorbance of the supernatant was measured at 532 nm using a spectrophotometer (Eppendorf). MDA content was determined using the method described previously (Heath and Packer, 1968). H_2_O_2_ content was measured by reacting it with 1 M potassium iodide and 100 mM potassium-phosphate buffer (pH 7.0). The reaction mixture was incubated in the dark for 1 h, and the absorbance was measured at 390 nm. The amount of H_2_O_2_ was calculated using a standard curve prepared using known concentrations of H_2_O_2_ (Alexieva et al., 2001).

SOD, CAT, APX, POD, and GR activities were determined using specific assay kits: SOD assay kit (cat. DG-SOD400, Dogen, Seoul, Korea), CAT assay kit (cat. DG-CAT400, Dogen), APX kit (MBS2602897, MyBioSource, San Diego, CA, USA), POD ELISA kit (MBS9313803, MyBioSource), and GR microplate assay kit (MBS8243195, MyBioSource). The optical density was measured at specific wavelengths using an automated plate reader (SpectraMax M2; Molecular Devices, San Jose, CA, USA). IAA and ABA levels were determined using ELISA kits, IAA ELISA kit (MBS269958, MyBioSource) and ABA ELISA kit (MBS703081, MyBioSource). The optical density was measured at specific wavelengths using an automated plate reader (SpectraMax M2; Molecular Devices).

### 2.6 RNA sequencing and analysis of differentially expressed genes

Total RNA was isolated in triplicates from control, drought, and rewatered plants’ leaves growing in the greenhouse. The RNeasy Plant Mini Kit (Qiagen, Hilden, Germany) or the Beniprep Super Plants RNA Extraction Kit from Invirustech (Gwangju, Korea) was used according to the manufacturer’s guidelines. The quality of the isolated RNA was assessed using a NanoDrop ND–1000 spectrophotometer (NanoDrop Technologies, Wilmington, DE, USA). DNA contamination in RNA samples was removed with RNase-free DNase (Promega, Madison, WI, USA). Double-stranded cDNA was synthesized using the cDNA EcoDryTM Premix (TaKaRa Bio, Shiga, Japan) with equal amounts of high-quality RNA from each sample. cDNA libraries were prepared using the Illumina TruSeq Standard RNA Prep Kit (catalog #RS-122-2103; Illumina, San Diego, CA, USA) and 24 samples were sequenced on an Illumina Novaseq 6000 platform (DNAlink, Inc., Seoul).

RNA-Seq data were processed using FastQC (version 0.11.2) (Andrews, 2010). Low-quality reads and reads containing adapter sequences were removed using the Trimmomatic tool (0.40) to obtain clean data (Bolger et al., 2014). Clean paired-end reads were aligned with the available reference genome of *Q. suber* (https://www.ncbi.nlm.nih.gov/datasets/genome/GCA_002906115.4/) using HISAT2 (2.1.0) (Kim et al., 2015). The number of reads mapped to each gene and the level of gene expression in the number of fragments per kilobase of transcript sequence per million sequenced base pairs (FPKM) were quantified using StringTie (1.3.4) (Pertea et al., 2015). Principal component analysis (PCA) based on Spearman’s correlation coefficient was performed as a correlation index between biological replicates by pairwise comparison of samples, and the PCA plot was generated using pcaExplorer (http://shiny.imbei.uni-mainz.de:3838/pcaExplorer/) by loading the count data matrix along with its metadata information (Marini and Binder, 2019). (**Supplementary Figure 1**). Differential expression analyses between the *Qa* and *Qp* samples were performed using the DESeq2 R package (2.11.38). Genes with a *p*-value of 0.05 were considered as DEGs in the comparative analysis. The criteria for identification of DEGs were as follows: |log2Fold Change| threshold of 2 (≥+2 = upregulated and ≤ -2 = downregulated) and false-discovery rate (FDR)<0.05. Transcriptome data were deposited in the NCBI Sequence Read Archive (accession number PRJNA1087462).

### 2.7 Functional annotation of DEGs

Using the BLASTX search database in the OmicsBox tool, the assemblies were aligned with the NCBI nonredundant protein sequence, and only the best homolog was selected. Gene Ontology (GO) mapping was performed using the Gene Ontology database (version 3.1). The assemblies were aligned with the NCBI nonredundant protein sequence using the BLASTX search database in the OmicsBox tool, and only the best homolog was selected. In addition, Kyoto encyclopedia of genes and genomes (KEGG) signaling pathway genes were mapped with the DEGs using gene ids in *Q. acutissima* and *Q. palustris*. GO keywords were assigned to the annotated sequences using the OmicBox tool to predict the functions of the different sequences and the encoded proteins.

### 2.8 Weighted gene co-expression network analysis

The WGCNA(v1.1.75-2) package in R was used to create co-expression networks (Langfelder and Horvath, 2008). WGCNA was used to import gene expression values and use an automatic network construction function blockwise to create coexpression modules. Modules having default values, with the exception of minModuleSize being 50, power being 14, and TOMType being unsigned. Hub TFs were chosen from significant modules identified by calculating the correlation coefficient between module eigengenes and physiological data (Huang et al., 2020). Cytoscape_3.9.1 was used to visualize the networks (Shannon et al., 2003).

### 2.9 Quantitative reverse-transcription PCR

Real-time quantitative reverse transcription PCR (qPCR) analysis was performed on a CFX96 Touch Real-Time PCR Detection System (BIO-RAD, Hercules, CA, USA) using the IQTM SYBR Green Supermix (BIO-RAD). The qPCR included an initial denaturation at 95°C for 30 s, followed by 38 cycles of denaturation at 95°C for 5 s and annealing/extension at 60°C for 34 s. Three independent biological replicates were used, with three technical replicates for each biological replicate. The relative transcript abundance was analyzed using the 2^-ΔΔCt^ method (Livak and Schmittgen, 2001). Actin and CACs were used as internal reference genes for normalization of the qPCR results (Marum et al., 2012) (**Supplementary Table 1**).

### 2.10 Statistical analysis

Analyses were conducted using one-way ANOVA with multiple comparisons using Tukey’s honestly significant difference test (HSD). A *p*-value<0.05 was considered significant. Values are presented as the means ± standard deviation (SD).

## 3 Results

### 3.1 Growth and physiological changes in response to drought and rewatering

**Figure 1:**
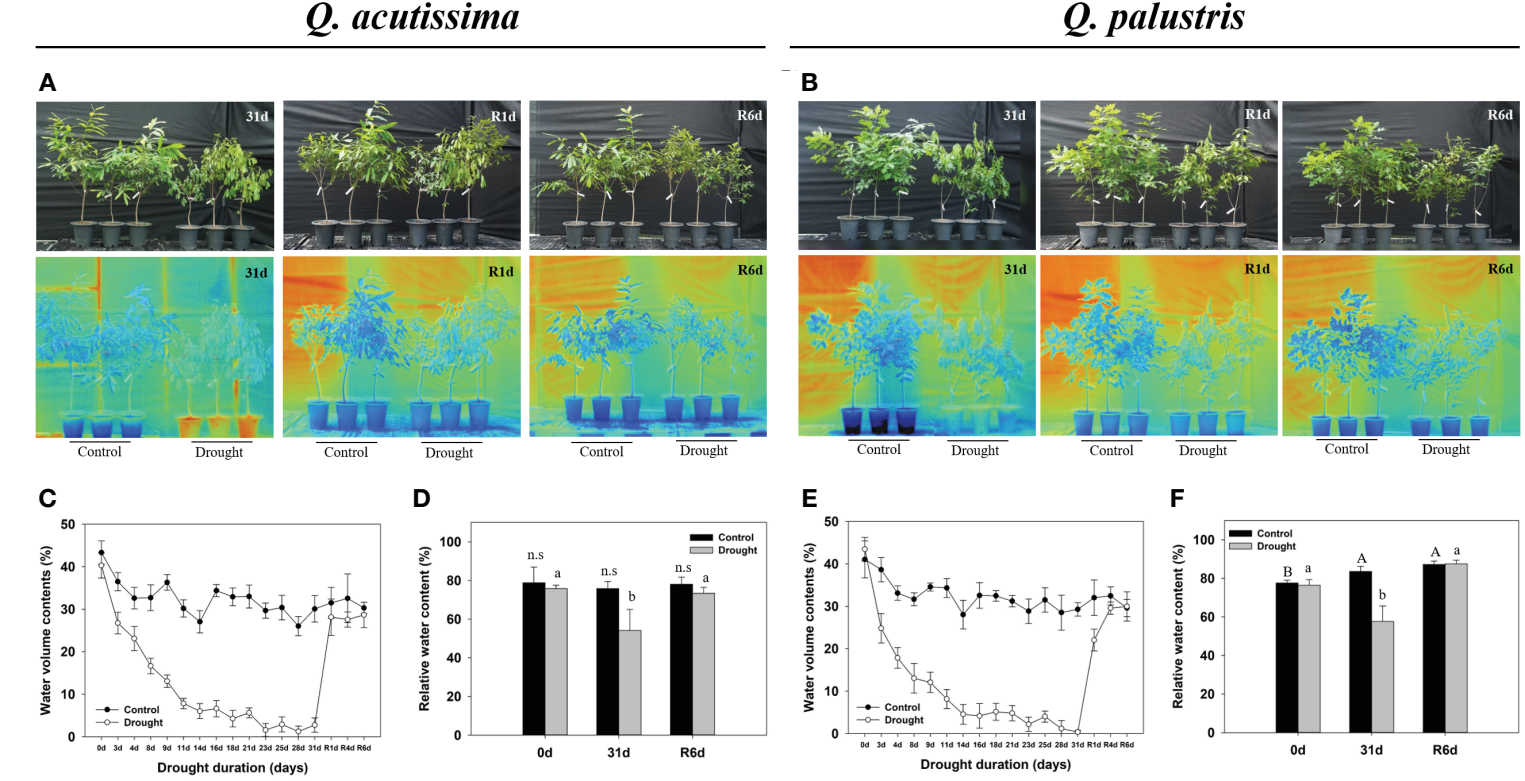
Representative phenotypes of *Quercus acutissima* and *Quercus palustris*. **(A, B)** Recovery of drought-stressed and control group seedlings after water resupply (upper); Infrared thermal images (bottom). **(C, E)** Volumetric water content of the soil in pots with drought-treated plants. **(D, F)** Relative water content (RWC) of the leaves. The values are the means ± SD (*n* = 10). Different uppercase letters and lowercase letters indicate significant differences (control: uppercase letters; drought: lowercase letters) (ANOVA with Tukey’s honestly significant difference test, *p*<0.05). **(A, C, D)**: *Q. acutissima*; **(B, E, F)**: *Q. palustris*).

**Figure 2:**
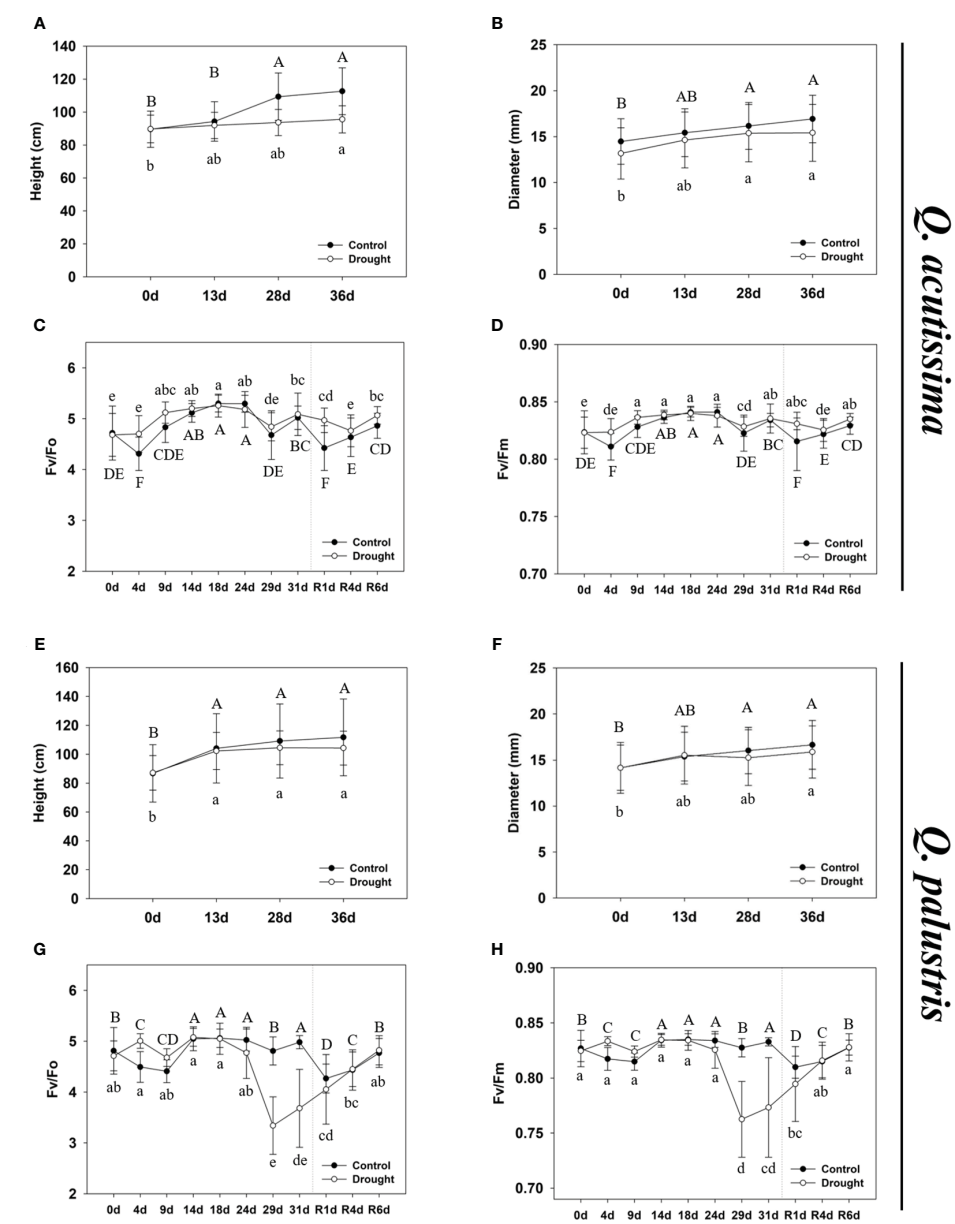
The growth phenotype and physiological changes in *Quercus acutissima* and *Quercus palustris*. **(A, E)** The effect of drought treatment and rewatering on shoot growth of plants. **(B, F)** The effect of drought treatment and rewatering on diameter of plants. **(C, G)** The Fv/Fo ratio. **(D, H)** The Fv/Fm ratio. The values are the means ± SD (*n* = 10). Different uppercase and lowercase letters indicate significant differences (control: uppercase letters; drought: lowercase letters) (ANOVA with Tukey’s honestly significant difference test, *p*<0.05). **(A-D)**: *Q. acutissima*; **(E-H)**: *Q. palustris*).

We recorded the changes in *Q. acutissima* and *Q. palustris* after the drought and rewatering (**Figures 1**, **2**). First, changes in withering, height, diameter, plant temperature, chlorophyll fluorescence, and chlorophyll content were evaluated. In *Q. acutissima*, the temperature after 31 days of drought treatment was 0.98°C higher in the drought than in the control, and after rewatering, the difference decreased to 0.37°C (**Figure 1A**). In contrast, *Q. palustris* showed a difference of 1.16°C between the control and drought treatment, and a difference of 1.21°C was maintained even after rewatering (**Figure 1B**). To investigate the physiological responses of oaks to water deprivation and recovery, phenotypic traits, including RWC, were evaluated at four time points: control, drought treatment, day 1 of rewatering (R1d), and day 6 of rewatering (R6d) (**Figures 1D, F**). During drought treatment and rewatering, the RWC of *Q. acutissima* and *Q. palustris* decreased by 54.2% and 57.7%, respectively. The RWC of drought-treated seedlings recovered to a level similar to that in control seedlings after rewatering. The height of *Q. acutissima* plants was significantly lowered after 28 d of drought treatment compared with that in the control (**Figure 2A**). The height of *Q. palustris* plants decreased after drought treatment compared with that in the controls (**Figure 2E**). After drought treatment and rewatering, the height did not increase significantly in both species. In terms of diameter, the growth of both species tended to be slightly delayed compared to that in the control group, but the difference was not statistically significant (**Figures 2B, F**). The other phenotypic traits analyzed were also strongly affected by water deprivation stress. For example, the leaves withered after 31d drought treatment. In contrast, well-watered control plants showed no sign of water shortage after 31d and had more abundant leaves. After rewatering for 24 h (1 d), the leaves of all drought-treated plants regained significant vigor and recovered to an extent similar to that in the control. However, the drought-treated plants that were rewatered were smaller than the control ones, and some leaves were still damaged after rewatering (**Figures 1A, B**).

The photosynthetic system is greatly affected by water deprivation stress. To determine the extent of drought-induced damage to the photosynthetic systems, we measured the changes in chlorophyll content and fluorescence response in stressed and rewatered plants. In *Q. acutissima*, no significant change in chlorophyll fluorescence, such as Fv/Fo and Fv/Fm, was observed after long-term drought treatment and rewatering, and there was no significant difference when compared with the control (**Figures 2C, D**). In contrast, in *Q. palustris*, chlorophyll fluorescence decreased in response to water deprivation stress, and a tendency for continued decrease was observed up to the 31st day of drought treatment, but it recovered after rewatering (**Figures 2G, H**). Changes in the chlorophyll content showed different trends in *Q. acutissima* and *Q. palustris* (**Table 1**). No significant change in the chlorophyll content of *Q. acutissima* was observed after drought treatment and rewatering. However, in *Q. palustris*, the chlorophyll content was clearly reduced when compared with that in the control or peaked during the drought treatment; it did not recover to normal levels after rewatering.

**Table 1:** Effects of drought stress treatment on photosynthetic pigments in *Quercus acutissima* Carruth. and *Quercus palustris* Münchh.

	Day	Treatment	mg/g FW ^(w)^				Chl a/b ^(w)^	Chl/Car ^(w)^
			Chl a	Chl b	Total Chl	Carotenoids		
*Quersuc acutissima* Carruth.	0	Control^(z)^	0.92 ± 0.04 ^N.S.^	0.21 ± 0.07 ^N.S.^	1.01 ± 0.07 ^N.S.^	0.22 ± 0.02 ^N.S.^	4.71 ± 1.97 ^N.S.^	4.70 ± 0.74 ^N.S.^
		Drought^(y)^	0.79 ± 0.21 ^n.s.^	0.15 ± 0.08 ^n.s.^	0.82 ± 0.29 ^n.s.^	0.19 ± 0.06 ^b^	6.44 ± 3.04 ^n.s.^	4.21 ± 0.59 ^n.s.^
	18	Control^(z)^	1.14 ± 0.11 ^N.S.^	0.25 ± 0.07 ^N.S.^	1.21 ± 0.18 ^N.S.^	0.26 ± 0.03 ^N.S.^	4.71 ± 1.14 ^N.S.^	4.64 ± 0.31 ^N.S.^
		Drought^(y)^	1.03 ± 0.01 ^n.s.^	0.19 ± 0.06 ^n.s.^	1.05 ± 0.08 ^n.s.^	0.25 ± 0.01 ^ab^	5.90 ± 1.69 ^n.s.^	4.24 ± 0.50 ^n.s.^
	24	Control^(z)^	1.16 ± 0.18 ^N.S.^	0.30 ± 0.03 ^N.S.^	1.30 ± 0.18 ^N.S.^	0.26 ± 0.05 ^N.S.^	3.91 ± 0.32 ^N.S.^	4.96 ± 0.26 ^N.S.^
		Drought^(y)^	1.04 ± 0.09 ^n.s.^	0.16 ± 0.01 ^n.s.^	1.03 ± 0.09 ^n.s.^	0.26 ± 0.03 ^a^	6.40 ± 0.41 ^n.s.^	4.04 ± 0.14 ^n.s.^
	31	Control^(z)^	0.94 ± 0.14 ^N.S.^	0.23 ± 0.02 ^N.S.^	1.13 ± 0.10 ^N.S.^	0.25 ± 0.03 ^N.S.^	4.67 ± 0.44 ^N.S.^	4.53 ± 0.23 ^N.S.^
		Drought^(y)^	1.02 ± 0.25 ^n.s.^	0.18 ± 0.11 ^n.s.^	1.04 ± 0.33 ^n.s.^	0.25 ± 0.04 ^a^	6.97 ± 2.95 ^n.s.^	4.04 ± 0.67 ^n.s.^
	R1	Control^(z)^	0.94 ± 0.15 ^N.S.^	0.16 ± 0.11 ^N.S.^	0.95 ± 0.25 ^N.S.^	0.23 ± 0.02 ^N.S.^	7.14 ± 2.84 ^N.S.^	4.12 ± 0.78 ^N.S.^
		Drought^(y)^	1.05 ± 0.15 ^n.s.^	0.20 ± 0.12 ^n.s.^	1.08 ± 0.27 ^n.s.^	0.25 ± 0.02 ^a^	8.11 ± 6.94 ^n.s.^	4.25 ± 0.81 ^n.s.^
	R4	Control^(z)^	1.01 ± 0.09 ^N.S.^	0.26 ± 0.10 ^N.S.^	1.14 ± 0.14 ^N.S.^	0.24 ± 0.04 ^N.S.^	4.25 ± 1.35 ^N.S.^	4.89 ± 1.17 ^N.S.^
		Drought^(y)^	0.97 ± 0.05 ^n.s.^	0.18 ± 0.03 ^n.s.^	1.02 ± 0.07 ^n.s.^	0.24 ± 0.01 ^ab^	5.35 ± 0.55 ^n.s.^	4.19 ± 0.20 ^n.s.^
	R6	Control^(z)^	0.82 ± 0.16 ^N.S.^	0.14 ± 0.05 ^N.S.^	0.83 ± 0.19 ^N.S.^	0.20 ± 0.04 ^N.S.^	6.45 ± 2.46 ^N.S.^	4.16 ± 0.49 ^N.S.^
		Drought^(y)^	1.04 ± 0.09 ^n.s.^	0.18 ± 0.08 ^n.s.^	1.07 ± 0.17 ^n.s.^	0.26 ± 0.01 ^a^	6.30 ± 2.24 ^n.s.^	4.09± ± 0.56 ^n.s.^
*Quercus palustris* Münchh.	0	Control^(z)^	0.84 ± 0.23 ^B^	0.29 ± 0.11 ^B^	1.06 ± 0.32 ^B^	0.19 ± 0.04 ^B^	2.97 ± 0.37 ^AB^	5.46 ± 0.63 ^N.S.^
		Drought^(y)^	0.92 ± 0.14 ^bc^	0.30 ± 0.10 ^abc^	1.19 ± 0.16 ^abc^	0.25 ± 0.03 ^abc^	3.27 ± 0.75 ^n.s.^	4.90 ± 1.14 ^n.s.^
	18	Control^(z)^	1.25 ± 0.05 ^A^	0.37 ± 0.08 ^AB^	1.48 ± 0.11 ^AB^	0.28 ± 0.02 ^A^	3.45 ± 0.52 ^A^	5.27 ± 0.78 ^N.S.^
		Drought^(y)^	1.18 ± 0.07 ^a^	0.40 ± 0.07 ^a^	1.49 ± 0.16 ^a^	0.27 ± 0.01 ^ab^	2.96 ± 0.34 ^n.s.^	5.44 ± 0.38 ^n.s.^
	24	Control^(z)^	1.18 ± 0.04 ^AB^	0.46 ± 0.01 ^A^	1.55 ± 0.03 ^AB^	0.25 ± 0.01 ^AB^	2.54 ± 0.05 ^B^	6.17 ± 0.15 ^N.S.^
		Drought^(y)^	1.18 ± 0.12 ^a^	0.38 ± 0.10 ^ab^	1.49 ± 0.23 ^a^	0.30 ± 0.03 ^a^	3.21 ± 0.61 ^n.s.^	5.03 ± 0.52 ^n.s.^
	31	Control^(z)^	1.26 ± 0.18 ^A^	0.47 ± 0.07 ^A^	1.60 ± 0.21 ^AB^	0.26 ± 0.03 ^AB^	2.71 ± 0.04 ^B^	6.13 ± 0.19 ^N.S.^
		Drought^(y)^	1.09 ± 0.15 ^ab^	0.36 ± 0.09 ^abc^	1.40 ± 0.20 ^a^	0.28 ± 0.05 ^ab^	3.14 ± 0.74 ^n.s.^	5.03 ± 0.76 ^n.s.^
	R1	Control^(z)^	1.31 ± 0.11 ^A^	0.44 ± 0.03 ^A^	1.63 ± 0.13 ^A^	0.30 ± 0.03 ^A^	2.98 ± 0.07 ^AB^	5.49 ± 0.27 ^N.S.^
		Drought^(y)^	0.96 ± 0.05 ^abc^	0.34 ± 0.07 ^abc^	1.25 ± 0.12 ^ab^	0.24 ± 0.01 ^bc^	2.92 ± 0.51 ^n.s.^	5.26 ± 0.67 ^n.s.^
	R4	Control^(z)^	0.99 ± 0.22 ^AB^	0.33 ± 0.09 ^B^	1.25 ± 0.28 ^AB^	0.24 ± 0.04 ^AB^	3.03 ± 0.28 ^AB^	5.28 ± 0.31 ^N.S.^
		Drought^(y)^	0.74 ± 0.17 ^c^	0.22 ± 0.07 ^c^	0.91 ± 0.22 ^c^	0.19 ± 0.03 ^c^	3.39 ± 0.30 ^n.s.^	4.71 ± 0.33 ^n.s.^
	R6	Control^(z)^	1.03 ± 0.09 ^AB^	0.37 ± 0.02 ^AB^	1.32 ± 0.10 ^AB^	0.23 ± 0.03 ^AB^	2.78 ± 0.20 ^B^	5.75 ± 0.33 ^N.S.^
		Drought^(y)^	0.76 ± 0.08 ^c^	0.23 ± 0.04 ^bc^	0.93 ± 0.11 ^bc^	0.19 ± 0.02 ^c^	3.36 ± 0.32 ^n.s.^	4.84 ± 0.28 ^n.s.^

^(z)^ Control; ^(y)^ Drought; ^(w)^ The values are the means ± SD (n = 3). Different uppercase and lowercase letters indicate significant differences, and “n.s” indicates not significant (ANOVA with Tukey’s honestly significant difference test, p<0.05).

### 3.2 Changes in drought stress indicators and metabolites in response to drought and rewatering

The effects of the treatment on metabolites, such as soluble sugars and starch, were investigated (**Table 2**). The glucose content in drought-treated *Q. acutissima* leaves increased significantly, peaking at 25d. After rewatering, the glucose levels recovered to pre-drought levels at R1d and declined further thereafter. Fructose levels were also increased, peaking at 25d and recovering to pre-drought levels at R4d. No changes in sucrose levels were observed under drought stress. Starch content decreased rapidly compared with that in the control during the drought treatment period and recovered to the control level after rewatering. The glucose content in *Q. palustris* increased after drought treatment, peaked on 25d, and gradually recovered after rewatering, but was only partially recovered on 6d of rewatering. Fructose levels increased from 19d after drought treatment and did not decrease or recover to the pre-drought levels, after rewatering. No significant changes were observed in the sucrose content. Starch content decreased more rapidly and severely in the drought stress treatment group; however, after rewatering, it recovered to a level similar to that in the control group.

**Table 2:** Effects of drought stress treatment on carbohydrate contents in *Quercus acutissima* Carruth. and *Quercus palustris* Münchh.

	Day	Treatment		mg/g FW ^(w)^			
			Glucose	Fructose	Sucrose	Starch	Total soluble sugar ^(w)^
*Quersuc acutissima* Carruth.	0	Control^(z)^	1.53 ± 0.23 ^A^	0.86 ± 0.31 ^A^	23.80 ± 2.95 ^A^	0.89 ± 0.25 ^AB^	132.66 ± 22.69 ^N.S.^
		Drought^(y)^	1.52 ± 0.29 ^bc^	0.96 ± 0.22 ^b^	21.30 ± 0.72 ^n.s.^	0.75 ± 0.24 ^b^	138.70 ± 32.35 ^ab^
	18	Control^(z)^	0.62 ± 0.23 ^B^	0.55 ± 0.21 ^AB^	20.80 ± 5.16 ^AB^	0.85 ± 0.31 ^AB^	117.34 ± 28.83 ^N.S.^
		Drought^(y)^	2.29 ± 0.90 ^abc^	1.82 ± 0.68 ^ab^	23.32 ± 0.98 ^n.s.^	0.34 ± 0.10 ^c^	152.09 ± 27.19 ^ab^
	24	Control^(z)^	0.94 ± 0.44 ^AB^	0.56 ± 0.16 ^AB^	14.70 ± 3.04 ^B^	0.40 ± 0.24 ^BC^	93.23 ± 16.23 ^N.S.^
		Drought^(y)^	3.33 ± 1.32 ^a^	2.50 ± 0.53 ^a^	20.87 ± 0.89 ^n.s.^	0.10 ± 0.02 ^c^	145.37 ± 17.06 ^ab^
	31	Control^(z)^	0.63 ± 0.19 ^B^	0.39 ± 0.20 ^AB^	16.37 ± 1.02 ^AB^	0.13 ± 0.07 ^C^	140.00 ± 37.74 ^N.S.^
		Drought^(y)^	2.69 ± 0.63 ^ab^	2.55 ± 0.98 ^a^	22.70 ± 4.36 ^n.s.^	0.18 ± 0.12 ^c^	167.99 ± 29.36 ^a^
	R1	Control^(z)^	0.68 ± 0.10 ^B^	0.38 ± 0.05 ^AB^	17.21 ± 0.15 ^AB^	0.84 ± 0.27 ^AB^	100.31 ± 12.91 ^N.S.^
		Drought^(y)^	1.65 ± 0.36 ^bc^	1.52 ± 0.24 ^ab^	22.42 ± 3.84 ^n.s.^	0.81 ± 0.15 ^b^	117.45 ± 6.45 ^b^
	R4	Control^(z)^	1.22 ± 0.19 ^AB^	0.67 ± 0.15 ^AB^	19.54 ± 3.18 ^AB^	1.52 ± 0.53 ^A^	132.86 ± 12.04 ^N.S.^
		Drought^(y)^	1.08 ± 0.38 ^c^	1.04 ± 0.40 ^b^	24.77 ± 1.12 ^n.s.^	1.31 ± 0.16 ^a^	112.74 ± 15.88 ^b^
	R6	Control^(z)^	1.11 ± 0.24 ^AB^	0.32 ± 0.18 ^B^	18.16 ± 2.33 ^AB^	1.34 ± 0.63 ^A^	123.37 ± 17.36 ^N.S.^
		Drought^(y)^	1.23 ± 0.46 ^c^	1.48 ± 0.48 ^ab^	23.00 ± 3.32 ^n.s.^	1.32 ± 0.14 ^a^	132.56 ± 27.78 ^a^
*Quercus palustris* Münchh.	0	Control^(z)^	0.84 ± 0.26 ^N.S.^	0.49 ± 0.17 ^B^	19.87 ± 1.72 ^A^	0.54 ± 0.21 ^BC^	71.45 ± 27.30 ^BC^
		Drought^(y)^	0.69 ± 0.04 ^c^	0.41 ± 0.32 ^b^	18.79 ± 2.16 ^n.s.^	0.55 ± 0.23 ^b^	96.96 ± 18.51 ^b^
	18	Control^(z)^	0.39 ± 0.06 ^N.S.^	0.46 ± 0.14 ^B^	15.72 ± 1.21 ^B^	0.23 ± 0.06 ^C^	69.28 ± 7.78 ^BC^
		Drought^(y)^	2.89 ± 0.29 ^ab^	1.63 ± 0.19 ^a^	19.64 ± 4.95 ^n.s.^	0.11 ± 0.04 ^c^	131.45 ± 28.41 ^b^
	24	Control^(z)^	1.02 ± 0.72 ^N.S.^	0.50 ± 0.14 ^B^	14.68 ± 2.78 ^B^	0.18 ± 0.01 ^C^	57.37 ± 9.12 ^BC^
		Drought^(y)^	4.20 ± 0.29 ^a^	2.61 ± 0.52 ^a^	19.68 ± 1.46 ^n.s.^	0.04 ± 0.01 ^c^	137.94 ± 21.21 ^b^
	31	Control^(z)^	0.79 ± 0.44 ^N.S.^	0.36 ± 0.05 ^B^	15.73 ± 1.54 ^B^	0.39 ± 0.46 ^BC^	47.75 ± 10.33 ^C^
		Drought^(y)^	3.93 ± 0.62 ^a^	2.07 ± 0.95 ^a^	17.64 ± 1.27 ^n.s.^	0.04 ± 0.02 ^c^	126.73 ± 3.88 ^b^
	R1	Control^(z)^	0.62 ± 0.14 ^N.S.^	0.87 ± 0.15 ^A^	15.63 ± 1.60 ^B^	0.31 ± 0.19 ^C^	98.45 ± 14.45 ^B^
		Drought^(y)^	3.57 ± 0.22 ^a^	2.26 ± 0.36 ^a^	20.02 ± 3.82 ^n.s.^	0.25 ± 0.04 ^c^	155.67 ± 7.77 ^b^
	R4	Control^(z)^	1.50 ± 0.82 ^N.S.^	0.61 ± 0.08 ^AB^	19.13 ± 3.32 ^A^	0.94 ± 0.06 ^AB^	98.77 ± 4.04 ^B^
		Drought^(y)^	2.81 ± 1.61 ^ab^	1.75 ± 0.61 ^a^	22.43 ± 6.48 ^n.s.^	1.04 ± 0.32 ^a^	162.65 ± 36.15 ^a^
	R6	Control^(z)^	0.71 ± 0.21 ^N.S.^	0.73 ± 0.15 ^AB^	16.42 ± 1.61 ^AB^	1.16 ± 0.68 ^A^	97.09 ± 14.64 ^B^
		Drought^(y)^	2.17 ± 0.69 ^b^	1.72 ± 1.03 ^a^	22.29 ± 3.69 ^n.s.^	0.92 ± 0.12 ^a^	124.13 ± 22.92 ^b^

^(z)^ Control; ^(y)^ Drought; ^(w)^ The values are the means ± SD (n = 3). Different uppercase and lowercase letters indicate significant differences, and “n.s” indicates not significant (ANOVA with Tukey’s honestly significant difference test, p<0.05).

**Figure 3:**
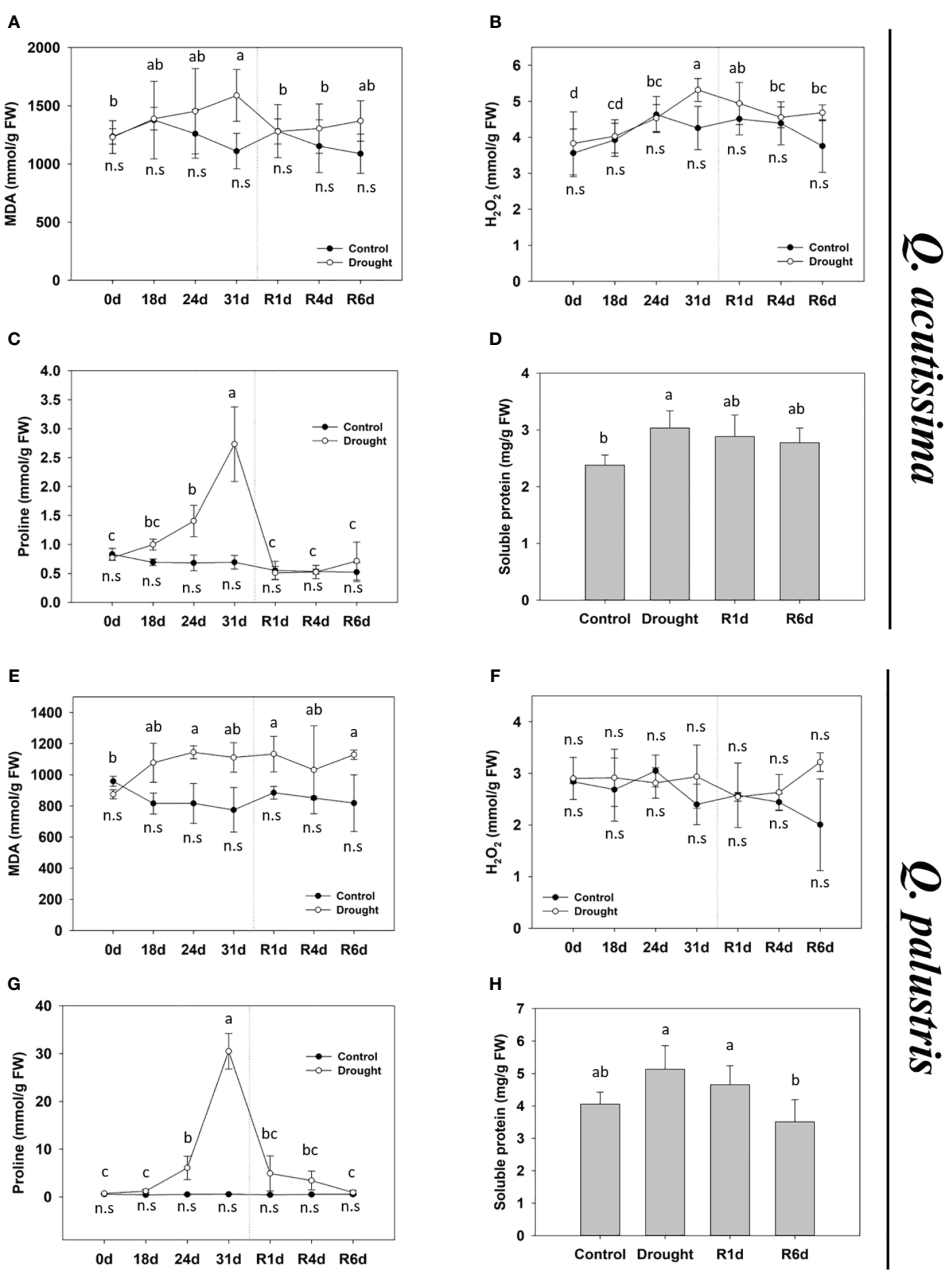
Effect of drought stress and rewatering on drought stress indicators in *Quercus acutissima* and *Quercus palustris*. **(A, E)** Malondialdehyde (MDA) content. **(B, F)** H_2_O_2_ content. **(C, G)** Proline content. **(D, H)** Soluble protein content. Different uppercase letters and lowercase letters indicate significant differences (control: uppercase letters; drought: lowercase letters) (ANOVA with Tukey’s honestly significant difference test, *p<* 0.05). **(A–D)**: *Q. acutissima*; **(E–H)**: *Q. palustris*).

We examined the changes in the stress indicators in response to drought stress. Changes in the MDA content differed between the two species. In *Q. acutissima*, the MDA content increased significantly after drought treatment and returned to normal after rewatering (**Figure 3A**). In contrast, the MDA content in *Q. palustris* increased after drought treatment and did not recover after rewatering (**Figure 3E**). The H_2_O_2_ content in *Q. acutissima* gradually increased after the drought treatment, peaked on 31d, and returned to normal levels after rewatering (**Figure 3B**). No significant changes in the H_2_O_2_ content were observed after drought treatment and rewatering in *Q. palustris* (**Figure 3F**). The proline content was greatly increased during drought treatment in both species and recovered to pre-drought levels after rewatering (**Figures 3C, G**). Both species showed similar increases in the water-soluble protein content of leaves under drought treatment (**Figures 3D, H**). After rewatering, *Q. acutissima* showed only partially decrease and recovered until R6d, whereas *Q. palustris* showed complete recovery to the pre-drought levels.

### 3.3 Changes in antioxidants and hormones in response to drought and rewatering

**Figure 4:**
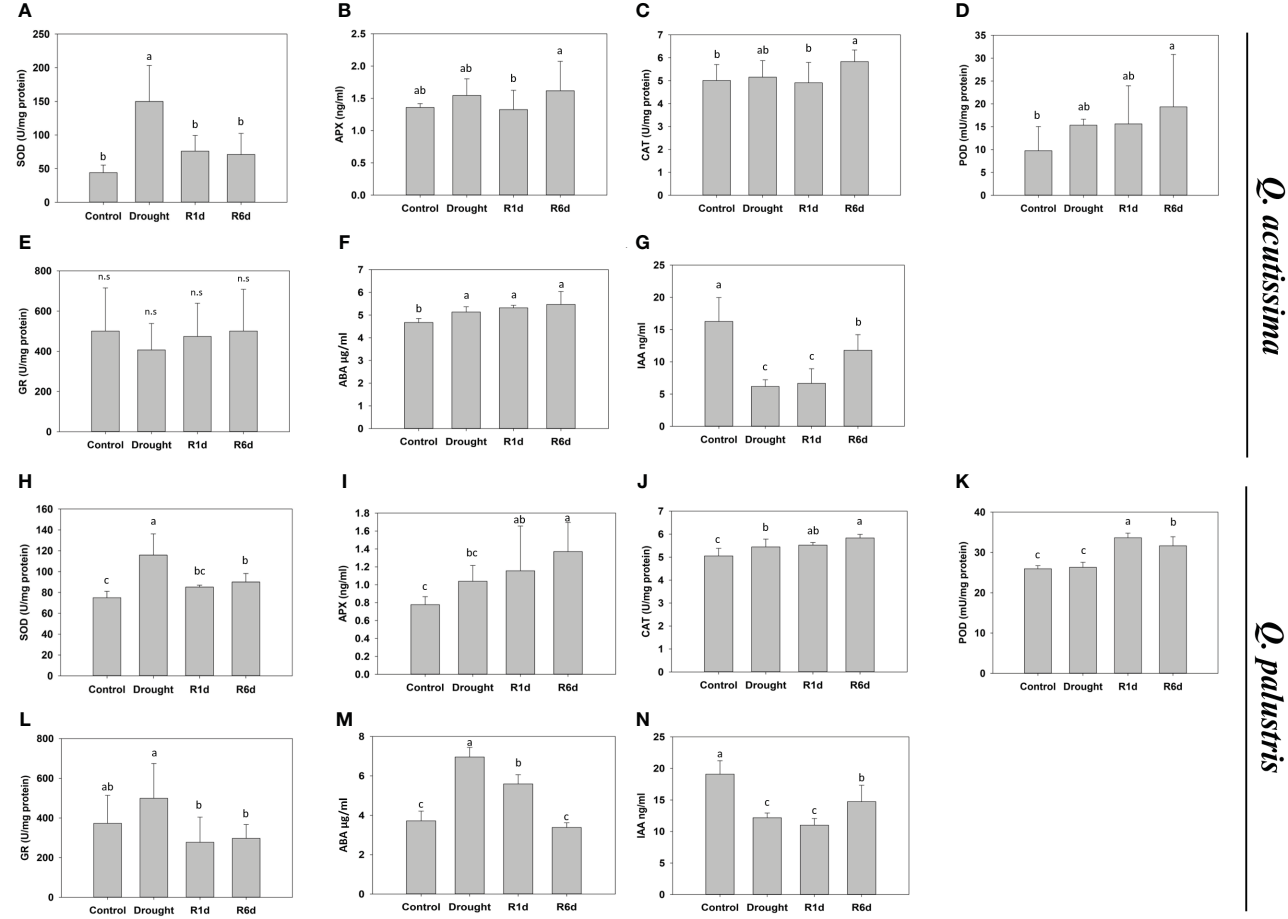
Effect of drought stress and re-watering on drought stress hormones and antioxidants in *Quercus acutissima* and *Quercus palustris*. **(A, H)** Superoxide dismutase (SOD) activity. **(B, I)** Ascorbate peroxidase (APX) activity. **(C, J)** Catalase (CAT) activity. **(D, K)** Peroxidase (POD) activity. **(E, L)** Glutathione Reductase (GR). **(F, M)** Abscisic acid (ABA) content. **(G, N)** Indole-3-acetic acid (IAA) content. Different lowercase letters indicate significant differences (ANOVA with Tukey’s honestly significant difference test, *p*<0.05). **(A–G)**: *Q. acutissima*; **(H–N)**: *Q. palustris*).

Antioxidants that remove excess ROS produced under stress are important factors in conferring drought tolerance to plants. Therefore, we measured changes in the activity of well-known antioxidant enzymes, such as SOD, APX, CAT, POD, and GR, during drought stress treatment and rewatering (**Figure 4**). The SOD activity increased during drought stress in both species and decreased after rewatering (**Figures 4A, H**). The SOD activity in *Q. acutissima* increased up to three times during drought treatment and almost recovered to the pre-drought levels one day after rewatering. In *Q. palustris*, it increased by approximately 1.5-times, and decreased only partially after rewatering. APX and CAT activities increased in *Q. palustris* during drought treatment and continued to increase after rewatering (**Figures 4I, J**). In contrast, in *Q. acutissima*, these values were only slightly increased at R6d (**Figures 4B, C**). The POD activity in *Q. acutissima* was significantly higher than that in the control at R6d (**Figure 4D**). In *Q. palustris*, it was greatly increased on the 1st day after rewatering and then partially decreased at R6d (**Figure 4K**). No significant difference in the GR activity was noted in *Q. acutissima*, whereas in *Q. palustris*, it slightly increased during the drought treatment and significantly decreased within 1 d after rewatering (**Figures 4E, L**). ABA and IAA play important roles in response drought stress. Therefore, we examined the changes in their content before and after drought and rewatering. In *Q. acutissima*, the ABA content increased under drought stress and did not recover after rewatering (**Figure 4F**). The IAA content was reduced to less than half during the drought treatment and partially increased upon rewatering (**Figure 4G**). In *Q. palustris*, the ABA content increased almost twice under drought stress conditions, and upon rewatering, it started to decrease from the 1st day and completely recovered to the level as in the control at R6d (**Figure 4M**). The IAA content was reduced by approximately 37% compared with that in the control during drought stress treatment and was partially increased at R6d after rewatering (**Figure 4N**).

### 3.4 Overview of transcriptome sequencing and mapping to the reference genome

**Figure 5:**
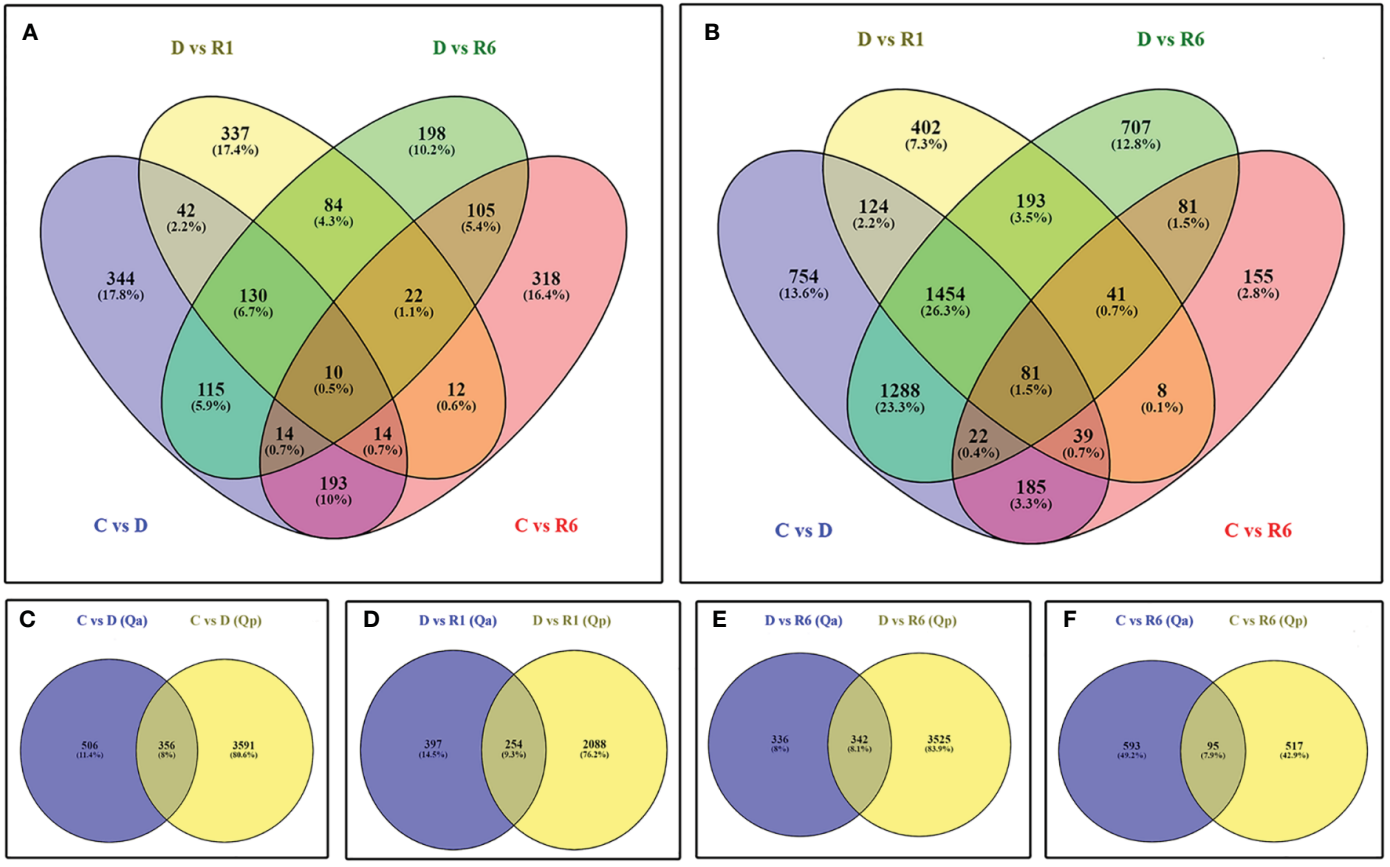
Identification of differentially expressed genes (DEGs) between the *Quercus acutissima* and *Quercus palustris* induced in response to drought and rewatering. Venn diagram displays **(A)** the number of DEGs across the comparisons Q vs. D, D vs. R1, D vs. R6, and C vs. R6 in *Q. acutissima*; **(B)** the number of DEGs across the comparisons Q vs. D, D vs. R1, Q vs. R6, and C vs. R6 in *Q. palustris*; and the number of DEGs between *Q. acutissima* and *Q. palustris* in the following comparisons **(C)** C vs. D, **(D)** D vs. R1, **(E)** D vs. R6, and **(F)** C vs. R6.

We generated ~722 million 101 bp pair-end reads, with approximately 30 million reads per sample. After quality trimming and filtering, approximately 670 million reads were retained with an average of 28 million reads per sample. The average GC percentage was 45% and the Q30 percentage was 96%. When mapping the RNA-seq reads to the reference genome, ~ 82.77% of the quality-filtered reads could be mapped (**Supplementary Table 2**). To gain insights into the differences between *Q. acutissima* (Qa) and *Q. palustris* (Qp) with control (C), drought-treated (D), rewatered at the 1^st^ day after drought (R1), and rewatered at the 6^th^ day after drought (R6), we extracted DEGs according to their differential expression levels, and based on C vs. D, D vs. R1, D vs. R6, and C vs. R6 comparisons, we identified 862 (330 upregulated; 532 downregulated), 651 (448 upregulated; 203 downregulated), 678 (421 upregulated; 257 downregulated), and 688 (320 upregulated; 368 downregulated) DEGs, respectively. In *Q. palustris*, C vs. D, D vs. R1, D vs. R6, and C vs. R6 comparisons revealed 3947 (2180 upregulated; 1767 downregulated), 2342 (967 upregulated; 1375 downregulated), 3867 (1578 upregulated; 2289 downregulated), and 612 (229 upregulated; 383 downregulated) DEGS, respectively (**Figure 5**). PCA and sample correlation study of this RNA seq study reveals that the sample groups are positively highly correlated and they segregated into different sample groups such as *Quercus acutissima and Q.palustris* both genewise and samplewise (**Supplementary Figures 1A, B, D**). The heatmap representing the pairwise correlation between different plant samples in also shown in **Supplmentary Figure 1C**.

### 3.5 Functional annotation of DEGs in response to drought and rewatering using GO enrichment analysis

**Figure 6:**
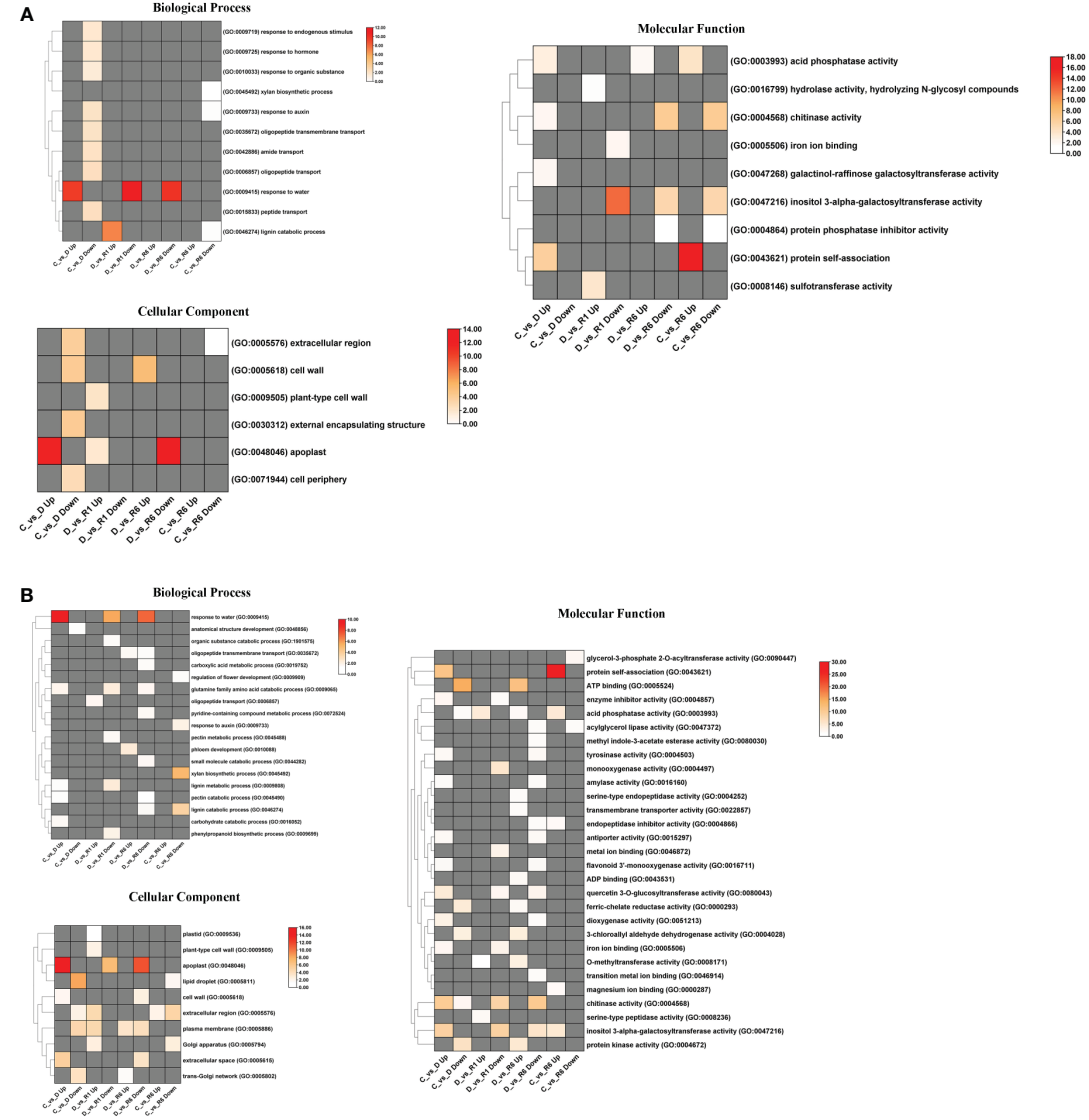
Heatmap representation of enriched GO terms annotated from the DEGs of *Quercus*. **(A)** denotes the GO terms enriched significantly (FDR ≤ 0.05) from the DEGs of *Q. acutissima*; **(B)** denotes the enriched significantly (FDR ≤ 0.05) from the DEGs of *Q. palustris*. The enriched GO categories such as Biological process, Molecular function and Cellular Component were shown individually. The differential colored boxes from white to red, indicate the numbers of genes enriched. The grey boxes represent the absence of significant GO terms in that sample.

In *Q. acutissima*, 496, 970, and 1130 unigenes were assigned to biological process (BP), molecular functions (MF), and cellular components (CC) GO categories, respectively, whereas in *Q. palustris*, 5422, 3446, and 2444 unigenes were assigned to the BP, MF, and CC categories, respectively. Four comparisons were performed for *Q. acutissima* and *Q. palustris*, viz. i) C vs. D, ii) D vs. R1, iii) D vs. R6, and iv) C vs. R6. GO enrichment was performed in Blast2GO using Fisher’s enrichment analysis. In *Q. acutissima*, upregulated DEGs in the C vs. D comparison were significantly enriched for “response to water” (GO:0009415) in the BP category, whereas this GO term was significantly enriched and downregulated upon rewatering. The upregulated DEGs in the C vs. D comparison were enriched for “apoplast” (GO:0048046) but this term was downregulated in the D vs. R6 comparison. In the MF category, “inositol 3-alpha-galactosyltransferase activity” was enriched. Another GO term, “protein self-association,” was enriched in the C vs. R6 comparison (**Figure 6A**). In *Q. palustris*, “response to water” was enriched for the DEGs upregulated in the C vs. D comparison and for the downregulated DEGs in the D vs. R1 and D vs. R6 comparisons. Notably, the “xylan biosynthetic process” (GO:0045492) and “lignin catabolic process” (GO:0046724) were enriched and downregulated in the C vs. R6 comparison. In the CC category, certain GO terms, such as “apoplast” (GO:0048046), “lipid droplet” (GO:0005811), “extracellular region” (GO:0005576), and “plasma membrane” (GO:0005886), were enriched. In the MF category, “Protein self-association” (GO:0043621) was enriched in the C vs. R6 and C vs. D comparisons (**Figure 6B**). The enrichment of GO terms in the upregulated and downregulated DEGs in *Q. acutissima* and *Q. palustris* is shown as bubble graph in **Supplementary Figure 2**.

### 3.6 Impact of drought stress and rewatering on the starch and sucrose metabolic pathway

**Figure 7:**
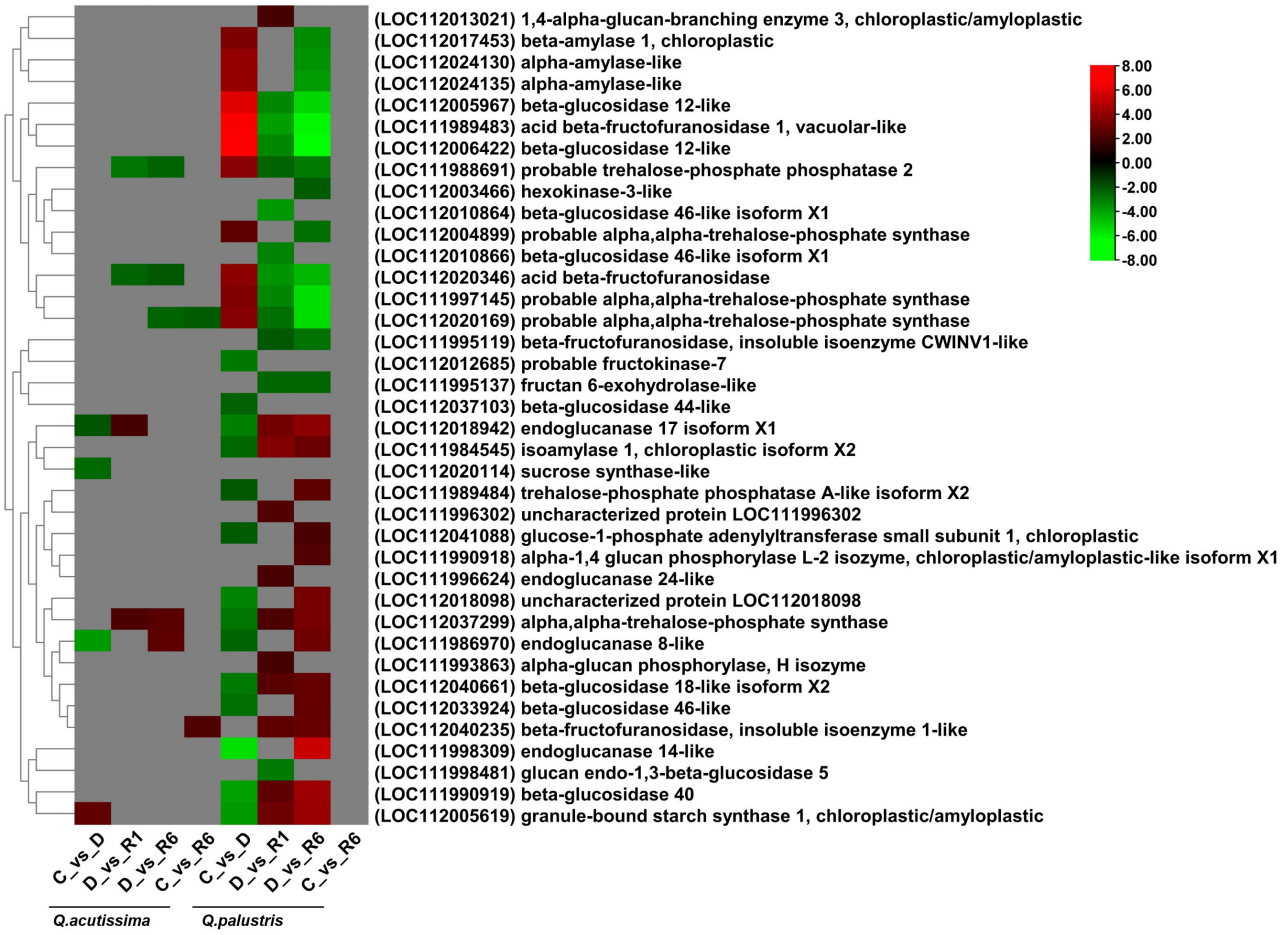
Heatmap showing differentially expressed genes (DEGs) related to the influence of the starch and sucrose pathway. Red indicates upregulated genes, green indicates downregulated genes, and gray indicates no significant differential expression of genes.

To understand the effect of drought stress and rewatering on starch and sucrose metabolic pathways, transcriptome data were analyzed. The DEGs regulated across the C vs. D, D vs. R1, D vs. R6, and C vs. R6 comparisons in *Q. acutissima* and *Q. palustris* are shown in **Figure 7**. In *Q.acutissima*, under drought conditions (C vs. D), three genes were downregulated and one was upregulated. The genes encoding sucrose synthase-like protein (LOC112020114), endoglucanase 8-like (LOC111986970), and endoglucanase 17 isoform X1 (LOC112018942) were downregulated. Simultaneously, granule-bound starch synthase 1 (LOC112005619) and chloroplastic/amyloplastic genes were upregulated in the C vs. D comparison. Genes related to probable trehalose-phosphate phosphatase 2 protein (LOC111988691) and acid beta-fructofuranosidase (LOC112020346) were downregulated, whereas the alpha, alpha trehalose phosphate synthase gene (LOC112037299) was upregulated in the D vs. R1 and D vs. R6 comparisons. After rewatering, two genes each were upregulated and downregulated in the D vs. R1 comparison, whereas three genes were downregulated and two were upregulated in the D vs. R6 comparison. Only one gene was upregulated and one was downregulated in the C vs. R6 comparison. Downregulated genes were related to probable alpha, alpha-trehalose-phosphate synthase (LOC112020169), whereas the upregulated gene was related to beta-fructofuranosidase, an insoluble isoenzyme 1-like protein (LOC112040235). In *Q. palustris*, genes encoding probable alpha, alpha-trehalose-phosphate synthase (LOC112004899), beta-amylase 1, chloroplastic (LOC112017453), probable alpha, alpha-trehalose-phosphate synthase (LOC111997145), probable alpha, alpha-trehalose-phosphate synthase (LOC112020169), probable trehalose-phosphate phosphatase 2 (LOC111988691), acid beta-fructofuranosidase (LOC112020346), alpha-amylase-like (LOC112024130), alpha-amylase-like (LOC112024135), beta-glucosidase 12-like (LOC112005967), acid beta-fructofuranosidase 1, vacuolar-like (LOC111989483), and beta-glucosidase 12-like (LOC112006422) were upregulated whereas those encoding endoglucanase 14-like (LOC111998309), beta-glucosidase 40 (LOC111990919), granule-bound starch synthase 1, chloroplastic/amyloplastic (LOC112005619), uncharacterized protein (LOC112018098), endoglucanase 17 isoform X1 (LOC112018942), beta-glucosidase 18-like isoform X2 (LOC112040661), probable fructokinase-7 (LOC112012685), alpha, alpha-trehalose-phosphate synthase (LOC112037299), beta-glucosidase 46-like (LOC112033924), isoamylase 1, chloroplastic isoform X2 (LOC111984545), endoglucanase 8-like (LOC111986970), beta-glucosidase 44-like (LOC112037103), glucose-1-phosphate adenylyltransferase small subunit 1, chloroplastic (LOC112041088), and trehalose-phosphate phosphatase A-like isoform X2 (LOC111989484) were downregulated in the C vs. D comparison. After rewatering, the upregulated genes in the C vs. D comparison were downregulated, and the downregulated genes were upregulated. In *Q. acutissima*, only a few genes responded to drought and rewatering, whereas in *Q. palustris* a larger number of genes related to starch and sugar metabolism were significantly down-regulated, especially after rewatering.

### 3.7 Impact of drought stress and rewatering on the glycolytic pathway

**Figure 8:**
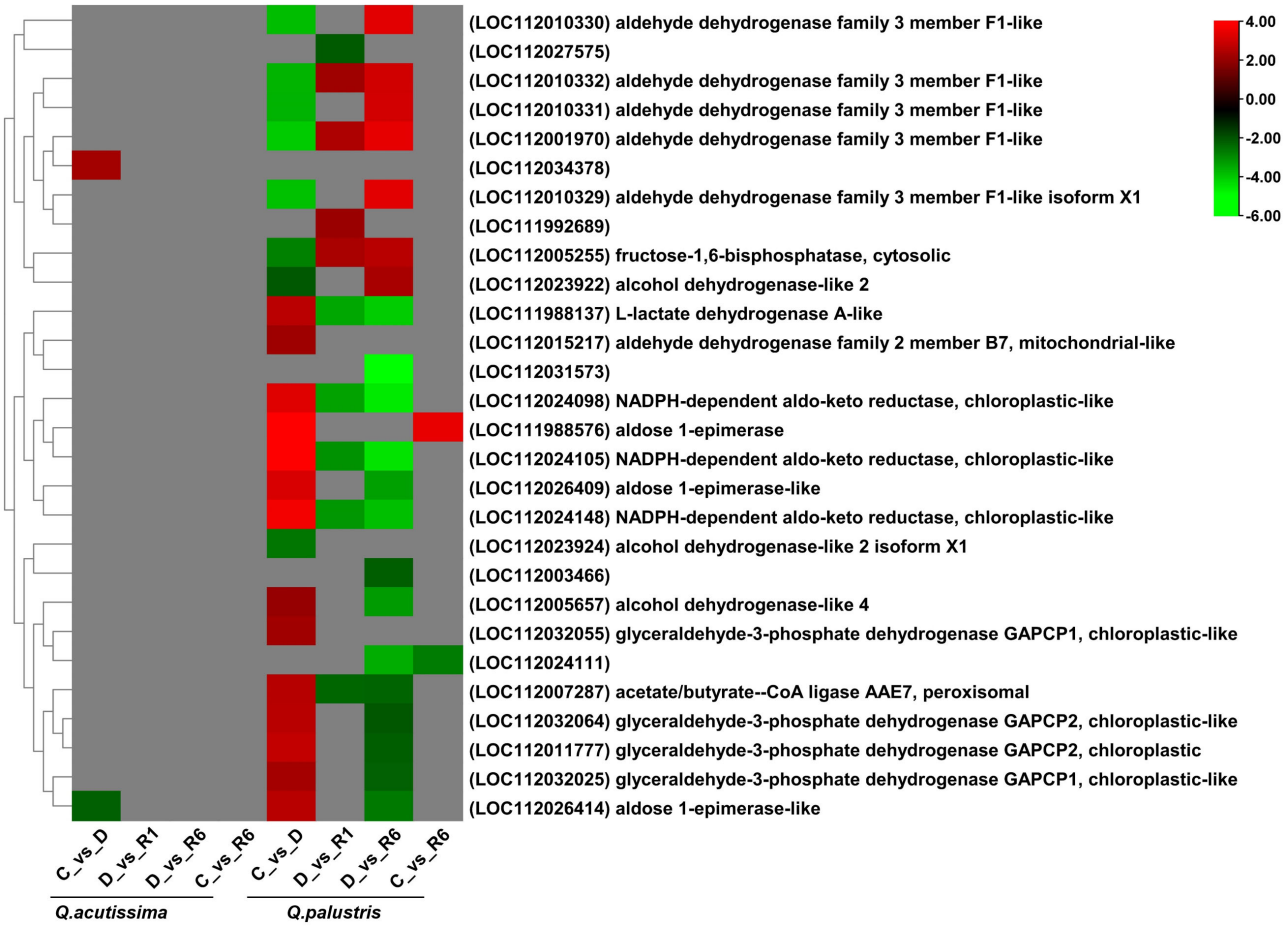
Heatmap showing differentially expressed genes (DEGs) related to the glycolytic pathway in *Quercus acutissima* and *Quercus palustris* under drought and rewatering conditions.

We also analyzed the effects of drought stress and rewatering on glycolysis-related genes among the DEGs in the two oak species. In *Q. acutissima*, only two genes related to the glycolytic pathway were significantly upregulated (**Figure 8**). A gene related to a putative protein (LOC112034378) was upregulated and that related to aldose 1-epimerase-like protein (LOC112026414) was downregulated. The importance of DEGs in the other comparisons was unknown whereas *Q. palustris* showed differential expression of genes encoding aldehyde dehydrogenase family 3 member F1-like (LOC112001970, LOC112010329, LOC112010330, LOC112010332, and LOC112010331), fructose-1,6-bisphosphatase, cytosolic protein (LOC112005255), alcohol dehydrogenase-like 2 isoform X1 (LOC112023924), alcohol dehydrogenase-like 2 (LOC112023922), which were significantly downregulated in both C vs. D comparisons. Downregulated genes were significantly upregulated in the D vs. R1 comparison. Other genes related to alcohol dehydrogenase-like 4 protein (LOC112005657), aldehyde dehydrogenase family 2 member B7, mitochondrial-like (LOC112015217), glyceraldehyde-3-phosphate dehydrogenase GAPCP1, chloroplastic-like (LOC112032025), acetate/butyr—e–CoA ligase AAE7, peroxisomal (LOC112007287), glyceraldehyde-3-phosphate dehydrogenase GAPCP2, chloroplastic-like (LOC112032064), aldose 1-epimerase-like (LOC112026414), L-lactate dehydrogenase A-like (LOC111988137), glyceraldehyde-3-phosphate dehydrogenase GAPCP2, chloroplastic (LOC112011777), aldose 1-epimerase-like (LOC112026409), NADPH-dependent aldo-keto reductase, chloroplastic-like (LOC112024098), NADPH-dependent aldo-keto reductase, chloroplastic-like (LOC112024148), aldose 1-epimerase (LOC111988576), and NADPH-dependent aldo-keto reductase, chloroplastic-like proteins (LOC112024105) were upregulated in the C vs. D comparison whereas these genes were significantly downregulated in the D vs. R6 comparison. In the C vs. R6 comparison, two genes encoding aldose 1-epimerase (LOC111988576) and a putative protein (LOC112024111) were upregulated and downregulated, respectively. We observed that compared to *Q. palustris*, only a few genes were regulated in *Q. acutissima*. In *Q. palustris*, most of the glycolytic related genes were upregulated during drought conditions whereas after rewatering those genes were significantly downregulated.

### 3.8 Changes in genes related to antioxidants induced by drought stress and rewatering

**Figure 9:**
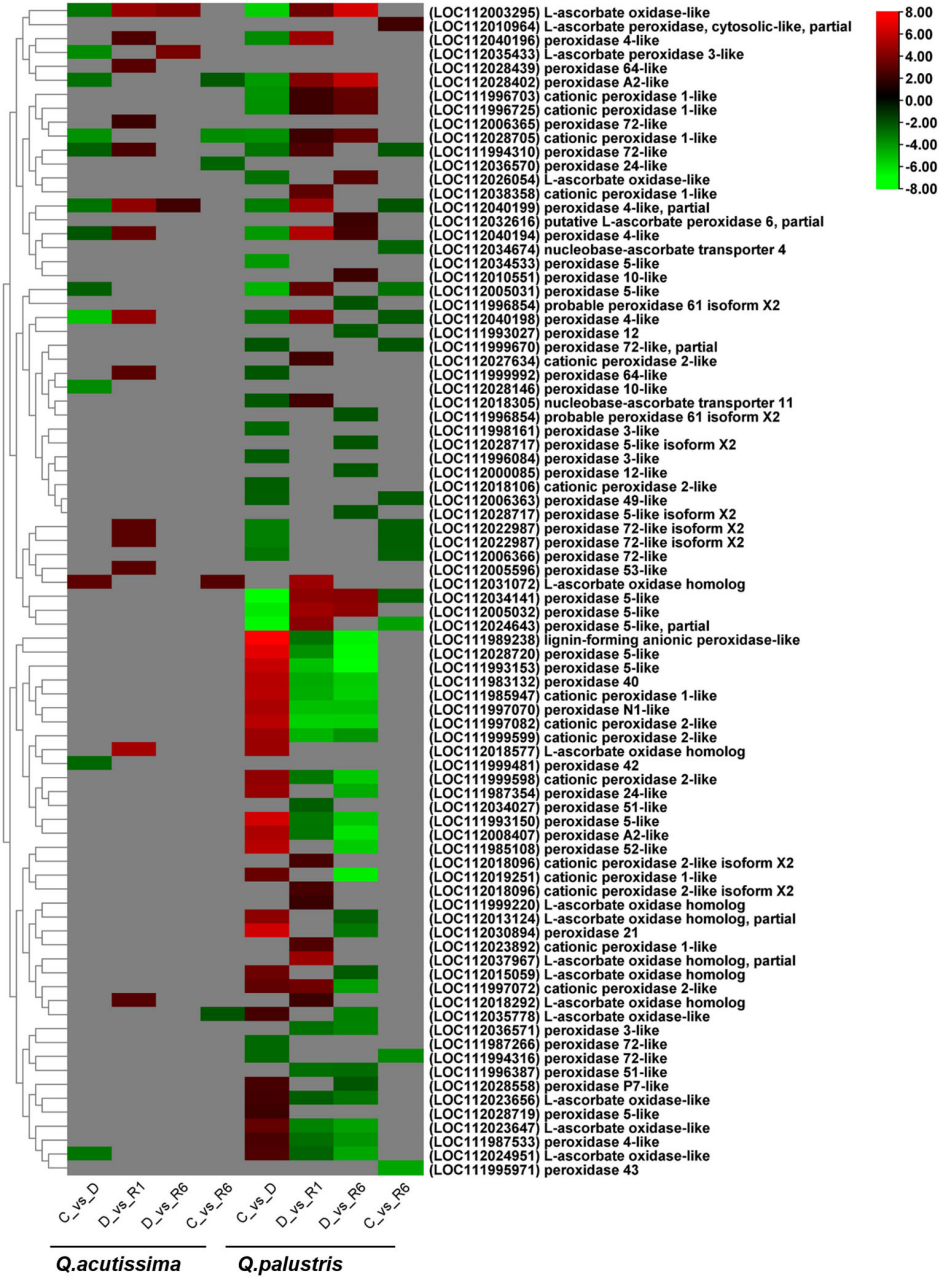
Heatmap showing antioxidant pathway-related differentially expressed genes (DEGs) regulated due to the impact of drought and rewatering conditions in *Quercus acutissima* and *Quercus palustris.*.

Next, we examined how drought and rewatering changed the expression of genes related to antioxidant enzymes using transcriptome analysis. In *Q. acutissima*, the number of genes regulated across the C vs. D, D vs. R1, D vs. R6, and C vs. R6 comparisons were 13, 14, 3, and 5, respectively (**Supplementary Figure 3**), whereas these numbers were 54, 43, 43, and 15, respectively for *Q. palustris*. In *Q. acutissima*, genes encoding peroxidase 4-like (LOC112040198), cationic peroxidase 1-like (LOC112028705), peroxidase 10-like (LOC112028146), L-ascorbate peroxidase 3-like (LOC112035433), L-ascorbate oxidase-like (LOC112003295), L-ascorbate oxidase-like (LOC112024951), peroxidase 4-like, partial (LOC112040199), peroxidase A2-like (LOC112028402), peroxidase 42 (LOC111999481), peroxidase 72-like (LOC111994310), peroxidase 5-like (LOC112005031), and peroxidase 4-like (LOC112040194) were significantly downregulated whereas that related to L-ascorbate oxidase homolog (LOC112031072) was significantly upregulated in the C vs. D comparison. The following genes encoding peroxidase 72-like (LOC112006365), peroxidase 72-like (LOC111994310), peroxidase 4-like (LOC112040196), L-ascorbate oxidase homolog (LOC112018292), peroxidase 64-like (LOC112028439), peroxidase 53-like (LOC112005596), peroxidase 72-like isoform X2 (LOC112022987), peroxidase 72-like isoform X2 (LOC112022987), peroxidase 64-like (LOC111999992), peroxidase 4-like (LOC112040194), peroxidase 4-like, partial (LOC112040199), peroxidase 4-like (LOC112040198), L-ascorbate oxidase-like (LOC112003295), and L-ascorbate oxidase homolog (LOC112018577) were upregulated in the D vs. R1 comparison. Only three genes encoding peroxidase 4-like, partial (LOC112040199), L-ascorbate peroxidase 3-like (LOC112035433), and L-ascorbate oxidase-like (LOC112003295) were upregulated in the D vs. R6 comparison. In the C vs. R6 comparison for *Q. acutissima*, genes encoding cationic peroxidase 1-like (LOC112028705), peroxidase 24-like (LOC112036570), peroxidase A2-like (LOC112028402), and L-ascorbate oxidase-like (LOC112035778) proteins were downregulated, whereas another L-ascorbate oxidase homolog (LOC112031072) was upregulated. After rewatering, most genes regulating L-ascorbate oxidase were upregulated (**Figure 9**). In *Q. palustris*, cationic peroxidase 1-like (LOC111996703, LOC111996725, LOC112028705, and LOC112018106), L-ascorbate oxidase-like (LOC112003295 and LOC112026054), nucleobase-ascorbate transporter 11 (LOC112018305), peroxidase 3-like (LOC111996084 and LOC111998161), peroxidase 49-like (LOC112006363), peroxidase 4-like (LOC112040194, LOC112040196, LOC112040198, and LOC112040199), peroxidase 5-like (LOC112005031, LOC112005032, LOC112034141, LOC112034533, and LOC112024643), peroxidase 64-like (LOC111999992), peroxidase 72-like (LOC111987266, LOC111994310, LOC111994316, and LOC112006366), peroxidase 72-like isoform X2 (LOC112022987, LOC112022987, and LOC111999670) and peroxidase A2-like (LOC112028402) were downregulated in the C vs. D comparison. The upregulated genes in the C vs. D comparison included those regulating peroxidase 5-like (LOC112028719), L-ascorbate oxidase-like (LOC112023656), L-ascorbate oxidase-like (LOC112035778), peroxidase P7-like (LOC112028558), peroxidase 4-like (LOC111987533), L-ascorbate oxidase-like (LOC112024951), cationic peroxidase 2-like (LOC111997072), L-ascorbate oxidase-like (LOC112023647), cationic peroxidase 1-like (LOC112019251), L-ascorbate oxidase homolog (LOC112015059), L-ascorbate oxidase homolog, partial (LOC112013124), cationic peroxidase 2-like (LOC111999598), peroxidase 24-like (LOC111987354), L-ascorbate oxidase homolog (LOC112018577), cationic peroxidase 2-like (LOC111999599), peroxidase N1-like (LOC111997070), peroxidase A2-like (LOC112008407), peroxidase 40 (LOC111983132), peroxidase 52-like (LOC111985108), cationic peroxidase 1-like (LOC111985947), cationic peroxidase 2-like (LOC111997082), peroxidase 5-like (LOC111993153), peroxidase 21 (LOC112030894), peroxidase 5-like (LOC111993150), peroxidase 5-like (LOC112028720), and lignin-forming anionic peroxidase-like (LOC111989238) (**Figure 9**).

Similarly, after rewatering, most of the peroxidases that were upregulated in the C vs. D comparison were downregulated in the D vs. R1 and D vs. R6 comparisons. Genes encoding proteins, such as L-ascorbate oxidase and cationic peroxidase-1 and -2 like, were upregulated in the D vs. R1 and D vs. R6 comparisons. In the C vs. R6 comparison, genes, such as peroxidase 43 (LOC111995971), peroxidase 5-like, partial (LOC112024643), peroxidase 72-like (LOC111994316), peroxidase 5-like (LOC112005031), peroxidase 5-like (LOC112034141), nucleobase-ascorbate transporter 4 (LOC112034674), peroxidase 72-like (LOC112006366), peroxidase 72-like isoform X2 (LOC112022987), peroxidase 72-like isoform X2 (LOC112022987), peroxidase 49-like (LOC112006363), peroxidase 4-like (LOC112040198), peroxidase 72-like (LOC111994310), peroxidase 72-like, partial (LOC111999670), peroxidase 4-like, partial (LOC112040199), and L-ascorbate peroxidase, cytosolic-like, partial (LOC112010964) were significantly downregulated in *Q. palustris*. In particular, peroxidase 43 (LOC111995971), nucleobase-ascorbate transporter 4 (LOC112034674), and L-ascorbate peroxidase, cytosolic-like, partial (LOC112010964) were downregulated only in the C vs. R6 comparison (**Figure 9**). In *Q. acutissima*, only a few genes were regulated compared to *Q. palustris*. In *Q. palustris* during drought conditions a high number of antioxidant genes were regulated. During restoration, the number of upregulated genes were significantly reduced while the number of downregulated genes were increased showing reduction in the antioxidant related genes.

### 3.9 Plant hormone signaling induced by drought stress and rewatering

Members of the plant hormone signaling pathway were tested for each comparison. In *Q. acutissima*, the genes encoding auxin-responsive protein SAUR21-like (LOC112004178), abscisic acid receptor PYL4-like (LOC112017749), and auxin-responsive protein SAUR22-like (LOC112004181) were downregulated in drought-stressed plants compared with that in control plants (C vs. D). Auxin-responsive protein-expressing genes were upregulated in the rewatered plants (D vs. R1 and D vs. R6), but few genes were regulated in the C vs. R6 comparison. After rewatering *Q. acutissima* (D vs. R1), the following genes related to probable protein phosphatase 2C 51 (LOC111984042), probable protein phosphatase 2C 51 (LOC112008236), probable protein phosphatase 2C 51 (LOC112008365), probable protein phosphatase 2C 24 (LOC112022545), probable protein phosphatase 2C 75 isoform X1 (LOC111987714), pathogenesis-related protein 1-like (LOC112017594), probable xyloglucan endotransglucosylase/hydrolase protein 23 (LOC112028408), and serine/threonine-protein kinase SAPK2-like proteins (LOC112039491) were downregulated whereas those encoding cyclin-D3-1-like (LOC112038380), auxin-responsive protein SAUR50-like (LOC112040183), probable xyloglucan endotransglucosylase/hydrolase protein 23 (LOC112028318), abscisic acid receptor PYL4-like (LOC112017749), auxin-responsive protein SAUR22-like (LOC112004181), cyclin-D3-2 (LOC112030063), auxin-responsive protein IAA18-like isoform X1 (LOC112003459), putative indole-3-acetic acid-amido synthetase GH3.9 (LOC112033575), auxin transporter-like protein 5 (LOC111995955), uncharacterized protein LOC111994735 isoform X1 (LOC111994735), auxin-responsive protein SAUR21-like (LOC112004178), and auxin-induced protein 15A-like protein (LOC111994736) were upregulated in the D vs. R1 comparison. A similar gene regulation pattern was observed in the D vs. R6 comparison, except that the pathogenesis-related protein 1-like (LOC112017594) and serine/threonine-protein kinase SAPK2-like (LOC112039491) were not regulated. In the treatment group, C vs. R6 of *Q. acutissima*, most of the defense-related proteins, such as ethylene-responsive transcription factor 1B (LOC111996147), ethylene-responsive transcription factor 1B-like (LOC112003251), pathogenesis-related protein 1-like (LOC112016185), basic form of pathogenesis-related protein 1-like (LOC112017586), basic form of pathogenesis-related protein 1-like (LOC112017588), cyclin-D3-2 (LOC112030063), and protein TIFY 10A-like (LOC112037230) were downregulated whereas the gene encoding histidine-containing phosphotransfer protein 2 (LOC111996700) was upregulated.

**Figure 10:**
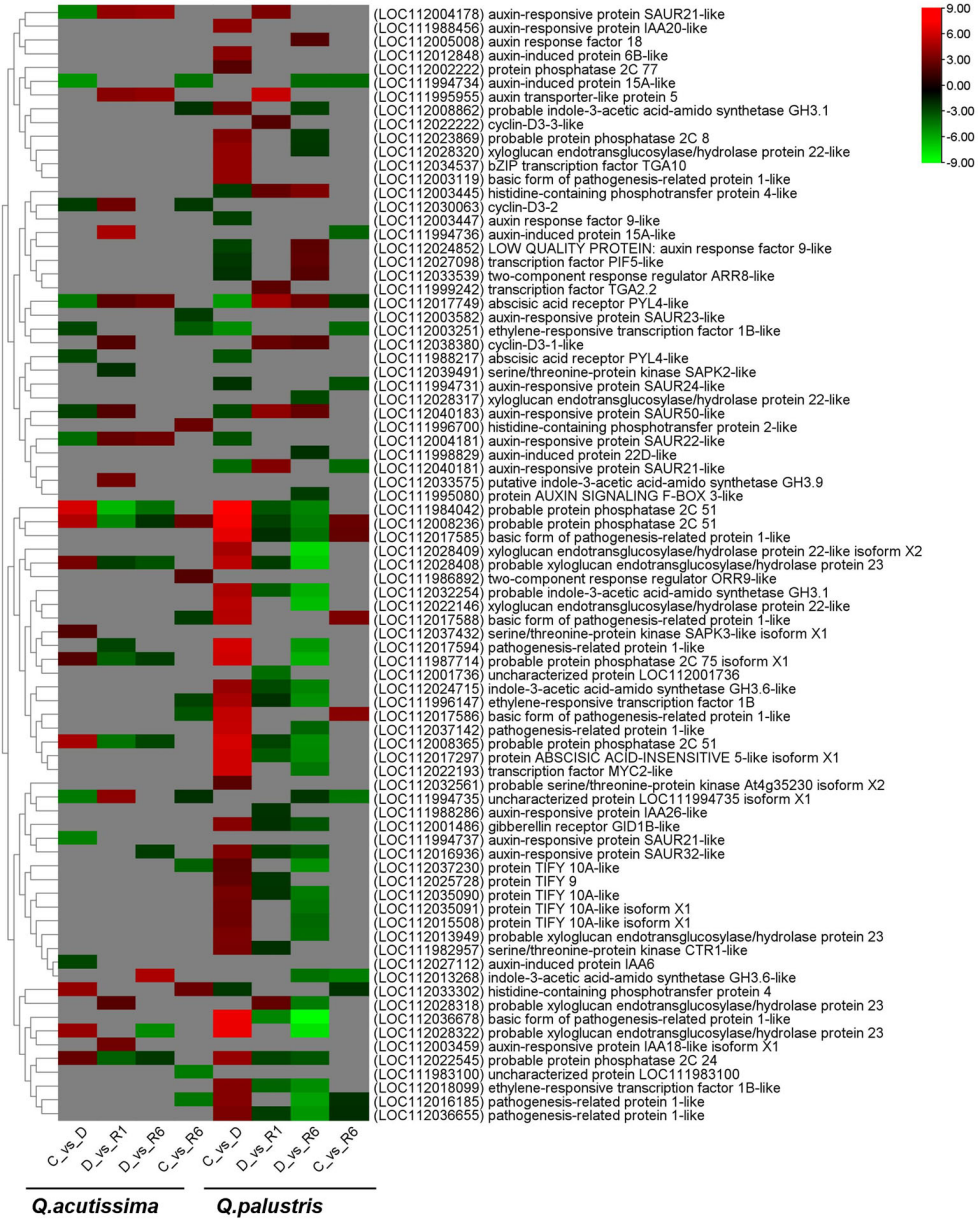
Heatmap showing plant hormone signaling pathway-related differentially expressed genes (DEGs) regulated due to the impact of drought and rewatering conditions in *Quercus acutissima* and *Quercus palustris*.

In *Q. palustris*, the following protein encoding genes were downregulated in the C vs. D comparison: auxin-induced protein 15A-like (LOC111994734), auxin-responsive protein SAUR21-like (LOC112004178), auxin-responsive protein SAUR21-like (LOC111994737), uncharacterized protein LOC111994735 isoform X1 (LOC111994735), abscisic acid receptor PYL4-like (LOC112017749), auxin-responsive protein SAUR22-like (LOC112004181), auxin-induced protein IAA6 (LOC112027112), abscisic acid receptor PYL4-like (LOC111988217), ethylene-responsive transcription factor 1B-like (LOC112003251), auxin-responsive protein SAUR50-like (LOC112040183), and cyclin-D3-2 (LOC112030063). The upregulated genes included serine/threonine-protein kinase SAPK3-like isoform X1 (LOC112037432), probable protein phosphatase 2C 75 isoform X1 (LOC111987714), probable protein phosphatase 2C 24 (LOC112022545), probable xyloglucan endotransglucosylase/hydrolase protein 23 (LOC112028408), histidine-containing phosphotransfer protein 4 (LOC112033302), probable xyloglucan endotransglucosylase/hydrolase protein 23 (LOC112028322), probable protein phosphatase 2C 51 (LOC112008365), probable protein phosphatase 2C 51 (LOC112008236), and probable protein phosphatase 2C 51 (LOC111984042) (**Figure 10**). The number of downregulated genes significantly increased during the late recovery stage (R6) compared with that in the early recovery stage (R1) in *Q. palustris*. Thirty genes were regulated in the D vs. R1 comparison, whereas 46 were regulated in the D vs. R6 comparison. Comparatively, 21 genes (LOC112028322, LOC112028409, LOC112022146, LOC111987714, LOC1120106185, LOC112017594, LOC112037230, LOC112022193, LOC112035091, LOC112013268, LOC112013949, LOC111994734, LOC112015508, LOC112037142, LOC112028317, LOC112008862, LOC111995080, LOC112023869, LOC112028320, LOC111994735, and LOC111998829) upregulated before recovery were downregulated after rewatering. In the treatment group, D vs. R6, the following genes were downregulated: probable protein phosphatase 2C 51 (LOC111984042 and LOC111987714), auxin-induced protein 15A-like (LOC111994734), an uncharacterized protein LOC111994735 isoform X1, protein AUXIN SIGNALING F-BOX 3-like (LOC111995080), ethylene-responsive transcription factor 1B (LOC111996147), auxin-induced protein 22D-like (LOC111998829), gibberellin receptor GID1B-like (LOC112001486), probable protein phosphatase 2C 51 (LOC112008236 and LOC112008365), probable indole-3-acetic acid-amido synthetase GH3.1 (LOC112008862), indole-3-acetic acid-amido synthetase GH3.6-like (LOC112013268), probable xyloglucan endotransglucosylase/hydrolase protein 23 (LOC112013949), protein TIFY 10A-like isoform X1 (LOC112015508), pathogenesis-related protein 1-like (LOC112016185, LOC112017594, LOC112036655, and LOC112037142), auxin-responsive protein SAUR32-like (LOC112016936), protein ABSCISIC ACID-INSENSITIVE 5-like isoform X1 (LOC112017297), basic form of pathogenesis-related protein 1-like (LOC112017585 and LOC112036678), ethylene-responsive transcription factor 1B-like (LOC112018099), xyloglucan endotransglucosylase/hydrolase protein 22-like (LOC112022146), transcription factor MYC2-like (LOC112022193), probable protein phosphatase 2C 24 (LOC112022545), probable protein phosphatase 2C 8 (LOC112023869), indole-3-acetic acid-amido synthetase GH3.6-like (LOC112024715), xyloglucan endotransglucosylase/hydrolase protein 22-like (LOC112028317), probable xyloglucan endotransglucosylase/hydrolase protein 23 (LOC112028318, LOC112028322, and LOC112028408), xyloglucan endotransglucosylase/hydrolase protein 22-like (LOC112028409 and LOC112028320), probable indole-3-acetic acid-amido synthetase GH3.1 (LOC112032254), protein TIFY 10A-like (LOC112035090 and LOC112037230), and their isoform X1 (LOC112035091). While accounting upregulated genes in the D vs. R6 comparison, genes, such as auxin response factor 18 (LOC112005008), two-component response regulator ARR8-like (LOC112033539), cyclin-D3-1-like (LOC112038380), LOW QUALITY PROTEIN: auxin response factor 9-like (LOC112024852), transcription factor PIF5-like (LOC112027098), auxin-responsive protein SAUR50-like (LOC112040183), abscisic acid receptor PYL4-like (LOC112017749), and histidine-containing phosphotransfer protein 4-like (LOC112003445), were abundant. In the C vs. R6 comparison, few genes, such as indole-3-acetic acid-amido synthetase GH3.6-like (LOC112013268), uncharacterized protein LOC111994735 isoform X1 (LOC111994735), auxin-induced protein 15A-like (LOC111994734), auxin-responsive protein SAUR21-like (LOC112040181), ethylene-responsive transcription factor 1B-like (LOC112003251), auxin-induced protein 15A-like (LOC111994736), auxin-responsive protein SAUR24-like (LOC111994731), abscisic acid receptor PYL4-like (LOC112017749), histidine-containing phosphotransfer protein 4 (LOC112033302), pathogenesis-related protein 1-like (LOC112016185), and pathogenesis-related protein 1-like (LOC112036655) were downregulated whereas other genes, including basic form of pathogenesis-related protein 1-like (LOC112017585, LOC112017586, and LOC112017588) and probable protein phosphatase 2C 51 (LOC112008236), were upregulated. Genes encoding defense-related proteins, such as pathogenesis-related protein 1-like, were downregulated in the C vs. R6 comparison in *Q. acutissima* but were upregulated in *Q. palustris*. In *Q. acutissima*, most of the plant hormone signaling genes were downregulated during drought condition while after recovering the DEGs were significantly reduced. In *Q. palustris*, the number of upregulated genes were higher than the downregulated genes during the drought condition. After rewatering, most of the plant hormone signaling genes were downregulated while a few were upregulated.

### 3.10 Comparison of DEGs between *Q. acutissima* and *Q. palustris*

Analysis of Venn diagrams for the upregulated and downregulated genes in the C vs. D comparisons showed that 356 genes were common. Most genes were downregulated, whereas only a few were upregulated (**Figures 5C–F**). The following top DEGs were regulated in common: thaumatin-like protein 1 (LOC112038887, LOC112038888, LOC112038876, LOC112038861, LOC112038862, and LOC111989390), linoleate 13S-lipoxygenase 2-1, chloroplast-like isoform X1 (LOC111990529), uncharacterized protein LOC112031465 (LOC112031465), uncharacterized protein LOC111995089 (LOC111995089), uncharacterized protein LOC112021193 (LOC112021193), membrane protein PM19L (LOC112032815), cysteine proteinase inhibitor B-like, partial (LOC112025257), early light-induced protein 1, chloroplastic-like (LOC111987464), protein MOTHER of FT and TFL1 (LOC112038587), cucumber peeling cupredoxin-like (LOC111984751), and probable protein phosphatase 2C 51 (LOC111984042) were upregulated whereas genes encoding putative ripening-related protein 1 (LOC112035773), chitinase-like protein 1 isoform X2 (LOC112000839), epidermis-specific secreted glycoprotein EP1-like (LOC112035353), uncharacterized protein LOC112006350 (LOC112006350), UDP-glycosyltransferase 73C5-like (LOC111984523), uncharacterized protein LOC112014150 (LOC112014150), NDR1/HIN1-like protein 1 (LOC112026165), E3 ubiquitin-protein ligase RHA2B-like (LOC112033407), peroxidase 4-like (LOC112040198), protein NRT1/PTR FAMILY 4.6-like isoform X1 (LOC112006249), phylloplanin-like (LOC112013165), probable sulfate transporter 3.4 (LOC112012045), serine/threonine-protein kinase phg2-like isoform X1 (LOC112012913), putative receptor-like protein kinase At4g00960 (LOC111986751), Not Available (LOC112022298), Not Available (LOC112033153), flowering-promoting factor 1-like protein 2 (LOC112017395), GDSL esterase/lipase At1g29670-like (LOC112005926), uncharacterized protein LOC111986110 (LOC111986110), GDSL esterase/lipase At5g45670-like isoform X1 (LOC112005567), ethylene-responsive transcription factor WIN1-like (LOC112002896), jacalin-related lectin 3-like isoform X1 (LOC111986119), and glutaredoxin-C11 (LOC112035836) were downregulated. The top downregulated gens included galactinol synthase 2-like (LOC112007268), cyclin-SDS (LOC112029982), late embryogenesis abundant protein 2-like (LOC111983933), membrane protein PM19L (LOC112032815), heavy metal-associated isoprenylated plant protein 27-like (LOC112008221), RNA-binding motif protein, X-linked-like-3 isoform X3 (LOC111983007), dehydrin Rab18-like (LOC112018691), late embryogenesis abundant protein 2-like (LOC111990835), cucumber peeling cupredoxin-like (LOC111984751), uncharacterized protein LOC111997339 (LOC111997339), RNA-binding motif protein, X-linked-like-3 (LOC111983863), galactinol synthase 2-like, partial (LOC112023339), and probable protein phosphatase 2C 51 (LOC111984042). In the D vs. R6 comparison, 342 genes were regulated in *Q. acutissima* and *Q. palustris*. Only 336 DEGs were specific to *Q. acutissima* whereas 3525 DEGs were regulated in *Q. palustris*. Most of the regulated genes mimicked those regulated in the D vs. R1 comparison. Only a few genes were regulated in the C vs. R6 comparison: 95 DEGs were common, whereas 593 and 517 DEGs were specifically expressed in *Q. acutissima* and *Q. palustris*, respectively (**Figure 5F**). The top common genes upregulated in the C vs. R6 comparison corresponded to acid phosphatase 1-like (LOC112008844), 22.0 kDa class IV heat shock protein-like (LOC111990377), acid phosphatase 1-like (LOC112008845), acid phosphatase 1-like (LOC112016015), probable terpene synthase 9 (LOC111983939), 17.5 kDa class I heat shock protein-like (LOC112017482), acid phosphatase 1-like (LOC112016014), flavonoid ‘\\’-monooxygenase-like (LOC111993015), 17.5 kDa class I heat shock protein-like (LOC112024436), and 22.0 kDa class IV heat shock protein-like (LOC112016302) whereas those encoding germin-like protein subfamily 1 member 16 (LOC112029728), auxin-induced protein 15A-like (LOC111994734), LRR receptor-like serine/threonine-protein kinase EFR, partial (LOC112021260), pathogenesis-related protein 1-like (LOC112016185), wall-associated receptor kinase 2-like (LOC112008483), phylloplanin-like (LOC112013165), glu *S. griseus* protease inhibitor-like (LOC112033155), glucan endo-1,3-beta-glucosidase-like (LOC112041147), EG45-like domain containing protein (LOC112015556), and ethylene-responsive transcription factor WIN1-like (LOC112002896) were downregulated. One of these genes, encoding Annexin D2-like protein, was significantly upregulated. Overall study on the DEGs during drought and recovery using rewatering shows that a majority of the plant hormone signaling genes and antioxidant pathway related genes were highly regulated compared to the genes from other two pathways, starch and sucrose metabolism and glycolytic pathway.

### 3.11 Co-expression network construction and identification of WGCNA modules

**Figure 11:**
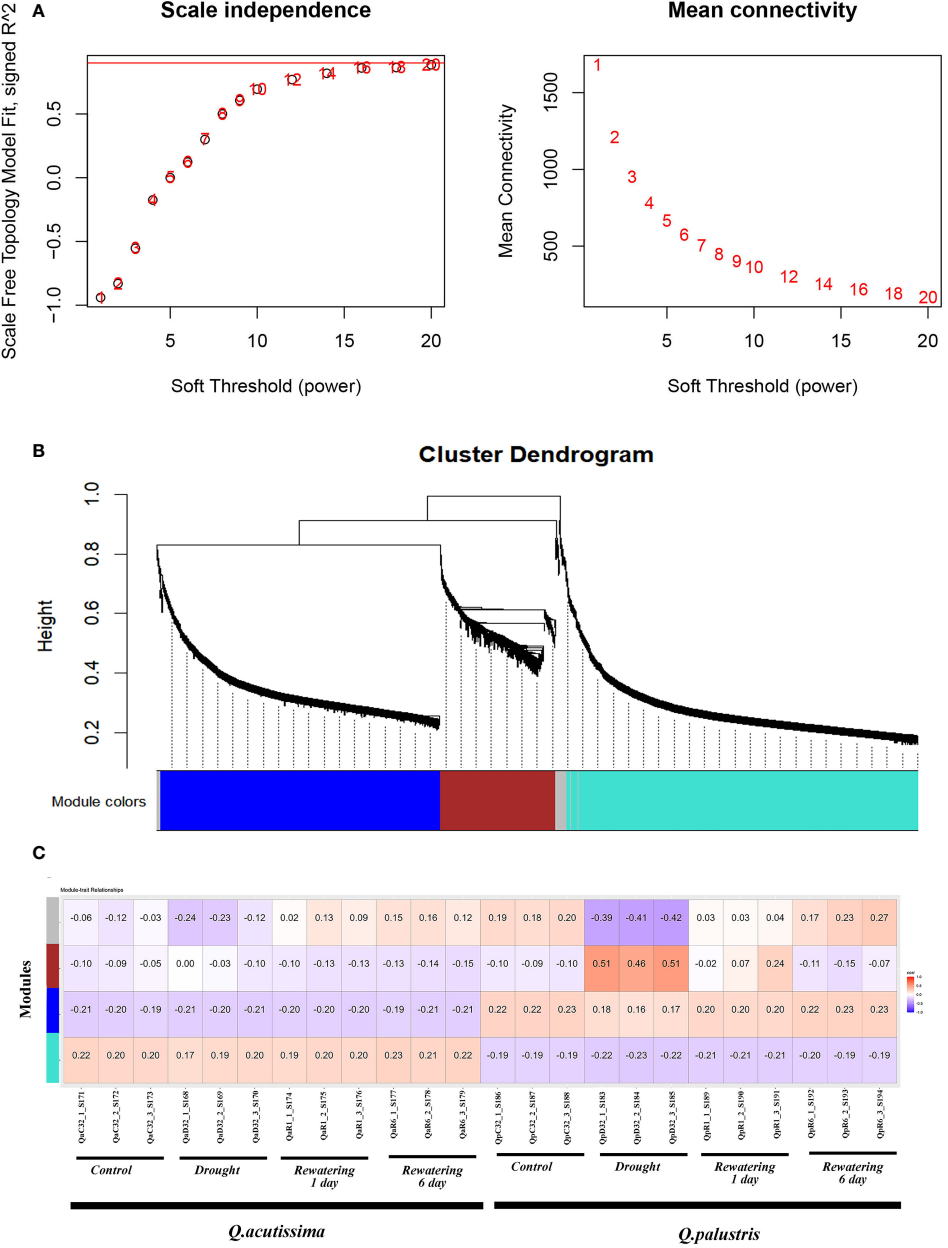
Module-trait correlation analysis and the WGCNA co-expression network. **(A)** Summary network indices (Y axis) as functions of the soft-Thresholding power (X-axis). The numbers in the plot indicates the corresponding soft thresholding power. Co-expression modules found using the Dynamic Tree Cut approach are displayed in **(B)** of the hierarchical cluster tree. Every leaf, or little vertical line, represents a gene. The dendrogram’s branches are colored-coded according to how closely they are related to one another to form modules. Genes exhibiting high levels of co-expression (correlation > 0. 75) were combined to form a single module, yielding a total of 4 modules. **(C)** WGCNA modules and physiological trait correlations. Every row represents a module. The columns represent characteristics of drought. The correlation coefficient between the attributes and the module is indicated by the color of each cell. Positive correlation is shown by red, and negative correlation is shown by blue. (The correlation coefficient is shown by the top number in the cell and the bottom one in parentheses represents the P value).

Gene co-expression network gene clustering and module cutting combined genes with similar expression patterns on the same branch. Each branch represented a co-expression module with different colors represented by different modules. During WGCNA, more than half of the samples were filtered, the expression pattern of 3232 genes out of 57626 were studied from Transcriptome sequencing was performed by WGCNA. According to the similarity of the expression patterns, we identified a total of 4 modules (**Figure 11**). The modules associated with different drought stress, the module-trait relationship was constructed (**Figure 11**). We identified that those genes in the ‘brown’ module is positively correlated with the drought stress whereas those genes in the ‘grey’ module was negatively correlated due to drought stress. Those genes in the module ‘turquoise’ was negatively correlated among *Q.palustris* compared to *Q.acutissima*, irrespective of drought conditions whereas in ‘blue’ module, the scenario is vice versa. A total of 1318, 1055, 437 and 72 genes were located in the WGCNA modules, turquoise, blue, brown and grey respectively, while remaining genes did not pass through the filters (**Figure 11**).

### 3.12 Validation of RNA-seq results using qPCR

**Figure 12:**
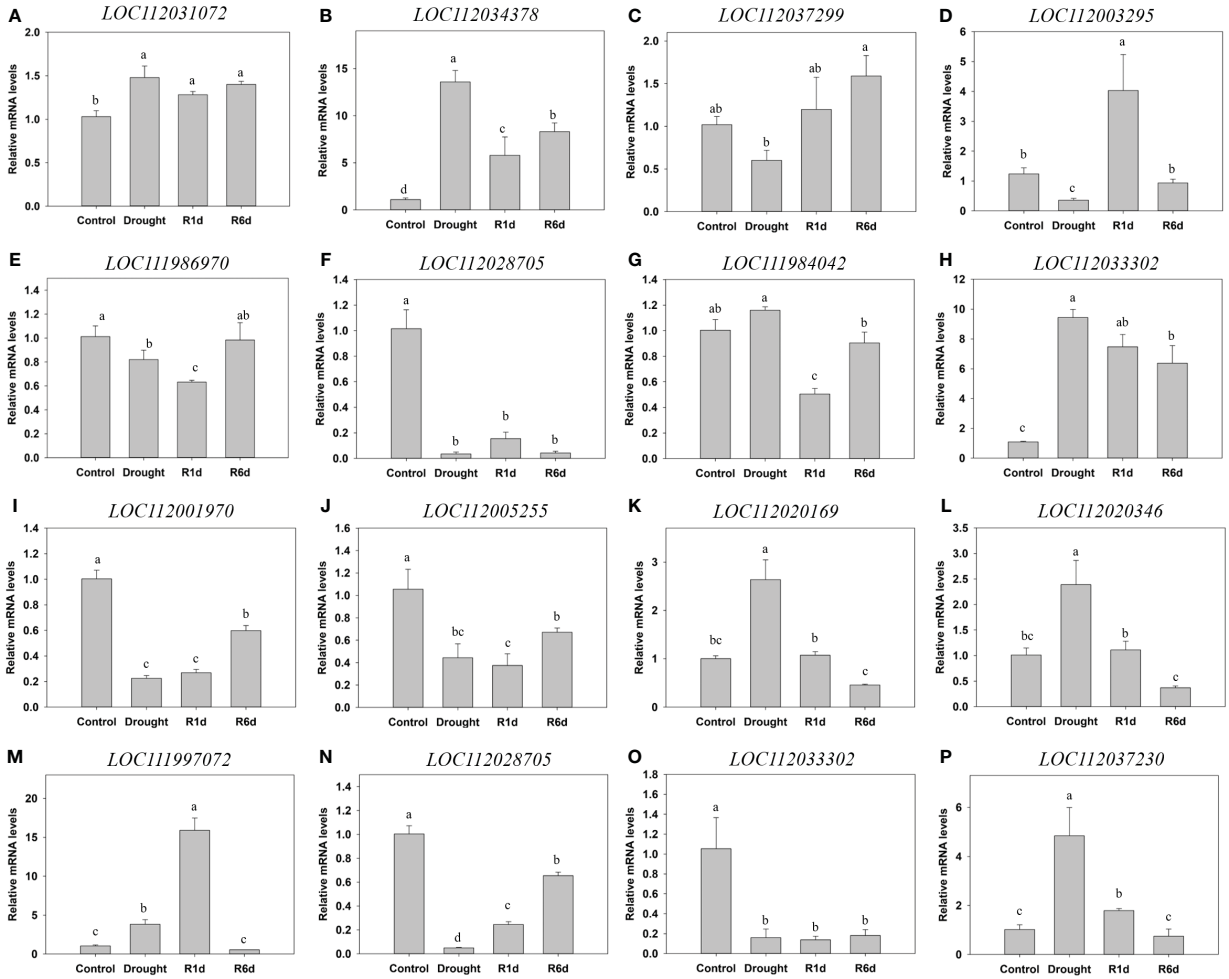
Validation of the differential expression of 16 genes using qPCR. **(A–H)** qPCR results for *Quercus acutissima*, and **(I)** to **(P)** qPCR results for *Quercus palustris*. **(A)** LOC112031072: L-ascorbate oxidase homolog. **(B)** LOC112034378: alcohol dehydrogenase-like. **(C)** LOC112037299: alpha, alpha-trehalose-phosphate synthase [UDP-forming] 1-like [KO:K16055] [EC:2.4.1.15 3.1.3.12]. **(D)** LOC112003295: L-ascorbate oxidase-like. **(E)** LOC111986970: endoglucanase 8-like. **(F)** LOC112028705: cationic peroxidase 1-like. **(G)** LOC111984042: probable protein phosphatase 2C 51 [KO:K14497] [EC:3.1.3.16]. **(H)** LOC112033302: histidine-containing phosphotransfer protein 4 [KO:K14490]. **(I)** LOC112001970: aldehyde dehydrogenase family 3 member F1-like [KO:K00128] [EC:1.2.1.3]. **(J)** LOC112005255: fructose-1,6-bisphosphatase, cytosolic [KO:K03841] [EC:3.1.3.11]. **(K)** LOC112020169: probable alpha, alpha-trehalose-phosphate synthase [UDP-forming] 9 [KO:K16055] [EC:2.4.1.15 3.1.3.12]. **(L)** LOC112020346: SAS, acid beta-fructofuranosidase . **(M)** LOC111997072: cationic peroxidase 2-like. **(N)** LOC112028705: cationic peroxidase 1-like. **(O)** LOC112033302: histidine-containing phosphotransfer protein 4 [KO:K14490]. **(P)** LOC112037230: protein TIFY 10A-like [KO:K13464]. Different lowercase letters indicate significant differences (ANOVA with Tukey’s honestly significant difference test, *p*<0.05).

To validate the DEGs identified using RNA-Seq, we selected 16 representative DEGs from categories including glycolysis, sucrose and starch metabolism, antioxidants, and hormones, and performed qPCR (**Figure 12**). In the glycolysis pathway in *Q. acutissima*, LOC112034378 was upregulated during drought and only partially decreased at R1d and R6d. In the sucrose and starch synthesis pathway, LOC112037299 expression decreased during drought and displayed a tendency to recover upon rewatering. Additionally, LOC111986970 decreased during drought and recovered in R6d. A gene associated with antioxidant enzymes, LOC112003295, was downregulated during drought, and was substantially upregulated at R1d, recovering to control levels at R6d. LOC112028705 exhibited significant downregulation after drought. LOC112031072 remained increased during drought. The hormone synthesis-related gene, LOC111984042, was upregulated during drought, downregulated at R1d, and recovered to the control levels at R6d. Additionally, LOC112033302 was substantially upregulated during drought and only partially decreased after rewatering, maintaining an upregulated state compared with that in the control. In *Q. palustris*, the glycolysis pathway-related genes, LOC112001970 and LOC112005255, were downregulated during drought, and showed partial recovery at R6d, indicating a tendency to remain downregulated compared with that in the control. LOC112020169 and LOC112020346 in the sucrose and starch synthesis pathway were significantly upregulated during drought and recovered to control levels after rewatering. The antioxidant enzyme-related gene, LOC111997072, was upregulated during drought, further upregulated until R1d, and recovered to control levels at R6d. LOC112028705 was markedly downregulated during drought and exhibited partial recovery upon rewatering. LOC112033302, in the hormone synthesis pathway, did not recover, maintaining a downregulated state after significant downregulation during drought, whereas LOC112037230 was substantially upregulated during drought and recovered to the control levels at R6d. Except for the difference in LOC112037230 between drought and R1d, the results of DEG analysis, qPCR, and RNA-Seq exhibited similar trends in most cases, confirming the reliability of the RNA-Seq analysis. The log_2_ fold change values obtained from RNA-seq expression exhibited a strong correlation with those from qPCR, as indicated by an R^2^ value of 0.831 (**Supplementary Figure 4**).

## 4 Discussion

### 4.1 Impact of drought stress on phenotypic traits

After subjecting the seedlings to 11 d drought, the soil moisture was below 10% (**Figures 1C, E**). In the 31-day treatment, it decreased to less than 3%. These results suggest that the seedlings were affected by water deficit. Despite being subjected to drought stress for the same period, the two species exhibited different phenotypic responses. *Q. acutissima* showed a definite decrease in tree growth, whereas *Q. palustris* showed a phenotype with extremely wilted leaves and only a slight decrease in growth (**Figures 2A, E**). These are presumed to be species-specific differences in the water deficit response. Leaf RWC is closely related to cell volume and provides a nuanced reflection of the balance between water supply and transpiration rate. It is a crucial indicator that influences a plant’s recovery from stress, thereby, impacting its yield and stability. RWC, especially under drought stress, is more informative than other water potential parameters. Normal RWC range from 98% in turgid, transpiring leaves to approximately 40% for severely desiccated leaves. For many plants, RWC at wilting is typically 60% to 70% (Lugojan and Ciulca, 2011). In this study, *Q. acutissima* and *Q. palustris* experienced water shortage due to drought treatment, and as a result, the RWC in both species decreased by approximately 55%, which indicates a wilting phenotype (**Figures 1D, F**). After rewatering, the normal state was restored. Therefore, it is presumed that the lack of water in plants due to drought stress and subsequent water replenishment were sufficiently induced. The decline in RWC during drought followed by recovery after rewatering suggests a dynamic response to water availability.

### 4.2 Photosynthetic efficiency and chlorophyll content

Drought stress influences plant growth, and its effects are contingent on the adaptation of plants to the duration and intensity of water deficit (Yang and Miao, 2010). We investigated the photochemical efficiency of PSII in response to drought using spectroscopic analysis of two oak leaves (**Figures 2C, D, G, H**). Water deficiency is widely recognized as a primary contributor to reduced PSII activity (Sagardoy et al., 2009). The two species showed completely different changes in Fv/Fo and Fv/Fm values. *Q. acutissima* showed no significant change, but *Q. palustris* showed a significant decrease in Fv/Fo and Fv/Fm during drought and recovered after rewatering. Fv/Fo reflects the ratio of the photochemical and nonphotochemical de-excitation fluxes of excited chlorophyll. These findings imply alterations in the rate of electron transport from PSII to primary electron acceptors with respect to density and size (Lu and Zhang, 2000). Fv/Fm signifies the maximum yield of primary photochemistry and overall PSII photosynthetic capacity. Fv/Fm less than 0.8 indicates inactivation or damage to the PSII reaction core. Therefore, the reduction in Fv/Fm in *Q. palustris* indicates damage to the photosynthetic system owing to drought. Analysis of PSII photochemical efficiency revealed distinct changes in the Fv/Fo and Fv/Fm values between *Q. acutissima* and *Q. palustris*.

Although influenced by factors, such as plant variety, duration, and growth stage, analyzing the leaf chlorophyll content remains a highly effective approach for assessing drought tolerance. No changes were observed in the chlorophyll content of *Q. acutissima* (**Table 1**). In *Q. palustris*, chlorophyll contents were slightly decreased after drought. Even after rewatering, the reduced chlorophyll content did not recover. The reduction in chlorophyll content caused by drought stress is commonly linked to oxidative stress and chlorophyll damage (Smirnoff, 1993). Stress affects chlorophyll levels, which are closely related to photosynthetic activity (Anjum et al., 2011), which is consistent with our observations in *Q. palustris*. Carotenoid levels remained steady in both species despite the drought, which is consistent with their reported antioxidant capacities (Deltoro et al., 1998). In summary, the response to drought was exclusively manifested as altered chlorophyll levels in *Q. palustris*, implying that it is more susceptible to drought stress than *Q. acutissima*.

### 4.3 Changes in metabolites and osmotic adjustment

Under drought stress, an increase in the level of soluble sugars can occur, which may help maintain their cell turgor by improving the water-holding and water-absorbing capacity (Guo et al., 2018). In the present study, the levels of total soluble sugars increased under drought (**Table 2**). However, after rewatering, the levels in *Q. acutissima* recovered to their original levels, whereas the change in *Q. palustris* was not recovered. Glucose and sucrose are osmolytes, whereas fructose is associated with secondary metabolite synthesis (Kaur and Gupta, 2005). Drought led to significant increases in the levels of glucose, fructose, and total soluble sugars, with the exception of sucrose and starch, in both species. Our findings align with those of a previous study indicating that drought generally leads to an increase in soluble sugar content (Rosa et al., 2009). Although it has been documented that many plants experience elevated sugar levels due to starch degradation during drought stress (Fischer and Höll, 1991), we observed only a slight increase in sucrose content in both species. In addition, there was no significant change in sucrose levels after rewatering. After rewatering, levels of glucose, total soluble sugars, and starch were completely recovered, and those of fructose were almost completely recovered in *Q. acutissima*; however, only starch levels were recovered, similar to that in the control, in *Q. palustris*. Therefore, *Q. palustris* responds more sensitively to drought stress than *Q. acutissima* and thus requires a longer recovery period. These results also show that *Q. palustris* requires more time to recover to normal levels after drought stress than does *Q. acutissima*.

In general, a decrease in the concentration of soluble proteins in plants is a representative feature of drought stress, although protein levels may increase rapidly in the immediate response to stress (Fischer and Höll, 1991). The accumulation of soluble proteins may be enhanced in plants under drought and confers drought tolerance (Chen and Plant, 1999). Under drought, levels of water-soluble proteins in both species increased and gradually normalized after rewatering (**Figures 3D, H**). Although *Q. palustris* had higher water-soluble protein content than *Q. acutissima*, it was phenotypically more susceptible to drought stress. The accumulation of proline, an osmolyte and a radical scavenger, in plants under drought is a drought-tolerance response (Yin et al., 2005). Both species showed a significant increase in proline during drought (**Figures 3C, G**). After rewatering, the levels were very rapidly recovered to normal. During drought, proline content also increased much more in *Q. palustris* than in *Q. acutissima* and then normalized after rewatering. Both species responded to drought and recovered by immediately and sensitively adjusting osmotic pressure by regulating proline concentration.

Osmotic balance in plants is maintained via the accumulation of compatible osmoprotectants by stabilizing cellular membranes and maintaining turgor (Sami et al., 2016). In the two oaks, the amounts of glucose, fructose, total soluble sugar, soluble proteins, and free proline increased under drought. Regarding resilience during rewatering, *Q. acutissima* tended to recover to normal levels. These results suggest that although *Q. palustris* responds sensitively to drought and increases osmoregulating substances, *Q. acutissima* has better drought tolerance and resilience owing to differences in regulatory ability.

In *Q. palustris*, genes related to alpha-amylase-like (LOC112024135 and LOC112024130) and beta-amylase 1 (LOC112017453) increased during drought and decreased upon rewatering (**Figure 7**) (Sun and Henson, 1991). Granule-bound starch synthase 1 (LOC112005619), a gene related to starch synthesis (Mérida et al., 1999), decreased and then recovered. These genes are related to starch-degrading enzymes, which is consistent with the increase in starch content compared with that in the control. Additionally, the increased expression of 1,4,-alpha-glucan-branching enzyme 3 (LOC112013021), which plays a role in structural transformation of starch (Ban et al., 2020) upon rewatering, was also associated with the recovery of starch levels. In *Q. palustris*, glucosidase-related genes (LOC112037103, LOC112010864, LOC111990919, LOC112033924, and LOC112040661), encoding cell wall lignification and starch hydrolytic enzymes (Minic, 2008), were downregulated, except for some genes (LOC112005967, LOC112006422, and LOC112010866). This appears to be related to changes in starch levels and growth of *Q. palustris* (**Table 2**). Notably, levels of genes related to endoglucanase, which converts cellulose polymers into small sugars and oligomeric polysaccharides (Glass et al., 2015), were decreased during drought but were recovered upon rewatering. This result is presumed to be related to sucrose levels, which showed little change in *Q. palustris* despite a decrease in starch during drought. LOC112004899, LOC111988691, LOC 112020169, LOC11997145, LOC111989484, and LOC112037299 are related to trehalose biosynthesis, which regulates the concentration of intracellular solvents during drought stress and contributes to the regulation of sucrose levels in higher plants (**Figure 7**) (Van Houtte et al., 2013). In *Q. acutissima*, changes in DEGs were observed upon drought and rewatering, which appears to be related to the results of our extraction experiments, in which sucrose levels continued to increase compared with those in the control (**Table 2**). In contrast, in *Q. palustris*, only minor changes in the results of extraction analysis were noted, but DEGs showed significant changes during drought and rewatering, and then tended to recover.

LOC112024098, LOC112024148, and LOC112024105 are related to NADPH-dependent aldo-keto reductase, which increased and then partially recovered (**Figure 8**). These are the first enzymes in the polyol pathway that converts glucose to sorbitol using NADPH as a cofactor (Singh et al., 2021). This is likely related to changes in glucose and fructose levels in *Q. palustris* (**Table 2**). Additionally, genes related to aldose-1-epimerase (LOC112026409, LOC112026414, and LOC111988576), which convert alpha-D-glucose to beta-D-glucose, showed a similar trend (Sheshukova et al., 2017). The expression of GAPCP (LOC112032055, LOC112032064, LOC112011777, and LOC112032025), a gene family that plays important roles in plant metabolic processes and is involved in stress response, increased during drought in *Q. palustris* and then decreased to some extent after rewatering, but remained higher compared than in the control (**Figure 8**) (Li et al., 2019b). These results indicated that the plant had not yet completely escaped the stressed state. The aldehyde dehydrogenase family (LOC112010331, LOC112010332, LOC112001970, LOC112010329, and LOC112010330) of enzymes, along with the cofactor, NAD+ or NADP+, convert aldehydes into carboxylic acids, NADH, or NADPH (Brocker et al., 2013). Compounds containing aldehyde functional groups are important intermediates in several catabolic and biosynthetic pathways. *Q. palustris* deteriorated during drought and did not fully recover thereafter, indicating that drought stress continued to affect glycolysis.

### 4.4 Oxidative damage and antioxidant enzymes

*Q. palustris* showed a significantly more wilted phenotype as drought progressed compared to *Q. acutissima*. MDA levels were slightly higher in *Q. acutissima* than in *Q. palustris* (**Figures 3A, E**). However, there was a difference in resilience after rewatering; levels in *Q. acutissima* recovered to normal, whereas those in *Q. palustris* did not recover until R6d. This also suggests that *Q. acutissima* has better drought tolerance than *Q. palustris*.

The H_2_O_2_ and SOD contents of *Q. acutissima* increased during drought and then recovered (**Figures 3**, **4**). In contrast, no change was observed in the H_2_O_2_ content in *Q. palustris*, but SOD and GR levels increased during the drought and normalized after rewatering (**Figures 3**, **4**). CAT and APX levels increased after drought and continued to increase after rewatering. SOD activity was significantly increased under drought, whereas POD activity tended to increase upon rewatering in both species, which is different from previous reports that SOD and POD activities are coregulated (Shigeoka et al., 2002). Previous studies have shown a different pattern than ours, noting increased POD activity under drought in various plants (Xiao et al., 2008). Our results showed that antioxidants control the increase in H_2_O_2_ through an appropriate response to drought at *Q. palustris*. However, when considering phenotypic changes or increases in MDA levels, it should be noted that cell damage is caused by a delay or lack of an antioxidant response or other factors. GR facilitates the reduction of GSH, which is involved in metabolic regulation and antioxidative processes using NADPH (Trivedi et al., 2013). Previous studies indicate the contribution of GR in enhancing drought stress tolerance (Romero-Puertas et al., 2006). Changes in GR activity were observed only in *Q. palustris*. No significant difference was noted between the control and drought groups; however, because the activity clearly decreased after rewatering, it might be the result of a reduction in stress factors (**Figure 4L**).

Differences in the responses of antioxidants among species suggest variations in the management of oxidative stress. Under drought stress conditions, the activities of APX, CAT, and GR, but not of SOD, showed better responsiveness in *Q. palustris* than to *Q. acutissima*; however, these results are contradictory to our other phenotypic observations. SOD primarily reduces cell damage by converting ROS, which damage cells more strongly, into H_2_O_2_, which has relatively weak activity. Therefore, the ability to express SOD and maintain its high activity is important for preventing cell damage. Considering these factors comprehensively, it is assumed that *Q. acutissima*, which has a good ability to initially remove ROS generated under stress through stronger SOD activity, has better durability and recovery than *Q. palustris*. Despite the tendency of antioxidant enzymes to increase at the beginning of the recognition of drought stress and then gradually decrease (Xiong et al., 2022), APX, CAT, POD, and GR responded more weakly in *Q. acutissima* than in *Q. palustris*. However, in this study, because we exposed the seedlings to drought for a relatively long period, we should consider whether the activities of these antioxidant enzymes were enhanced and then decreased, or whether the recognition of drought and the action of antioxidant enzymes were delayed. Notably, only the MDA levels in *Q. acutissima* recovered to the control level after rewatering. As a result, *Q. palustris* has poor tolerance and resilience under water stress, despite maintaining a higher composition and inductive activity of antioxidants, except for SOD, than does *Q. acutissima*. Our results show that antioxidants control the increase in H_2_O_2_ through an appropriate response to drought in *Q. palustris*. However, considering the phenotypic changes and increased MDA levels, cell damage might be due to a lack of control over other ROS.

In the transcriptome analysis, many DEGs related to antioxidants were identified, most of which were related to L-ascorbate oxidase or peroxidase (**Figure 9**). L-Ascorbic acid, commonly known as vitamin C, serves as a crucial redox buffer and a cofactor for enzymes that regulate various metabolic processes, including photosynthesis and hormone biosynthesis. In *Q. acutissima*, the expression of most of the genes related to L-ascorbate oxidase decreased during drought but showed a tendency to recover upon rewatering. However, only LOC112031072 maintained its upregulated expression. The peroxidase-related genes exhibited similar expression patterns. In particular, many peroxidase-related genes maintained reduced expression that did not recover after rewatering. Additionally, the remaining genes showed increased expression during drought. Upon rewatering, their levels decreased and recovered to the pre-drought levels. In *Q. acutissima*, cationic peroxidase genes related to POD showed increased expression in the D vs. R1 comparison, whereas in *Q. palustris*, genes with increased and decreased expression were observed. These genetic changes appear to have influenced the changes in POD levels in the oaks. In this study, we observed changes in the activities and expression of antioxidant activities, such as APX, CAT, and POD, during drought and recovery.

### 4.5 Hormonal regulation under drought stress

In the present study, *Q. palustris* showed a reciprocal interaction between IAA and ABA concentrations (**Figures 4M, N**). In contrast, ABA and IAA levels in *Q. acutissima* did not recover to pre-drought levels even after rewatering (**Figures 4F, G**). These results indicate an association between maintenance of high ABA levels and a substantial decrease in IAA levels, which likely contributed to the decline in shoot growth of *Q. acutissima* even after drought. In response to drought, IAA plays a crucial role in facilitating plant adaptation to drought. Recent studies have emphasized that alterations in auxin homeostasis under abiotic stress can affect ABA synthesis (Du et al., 2013). The equilibrium between auxin and ABA homeostasis has been identified as a pivotal factor in the diverse stress responses in *Arabidopsis*. In this study, IAA decreased significantly under drought in both the species and tended to increase again after recovery from stress to some extent on the 6th day after rewatering. This trend suggests that the plants entered a recovery period with an increase in antioxidants, such as CAT, APX, and POD, at R6d.

Both oaks showed changes in small auxin up-regulated RNA (SAUR)-related genes (LOC112004178, LOC111994731, LOC11240183, LOC112004181, and LOC112016936) in response to drought and rewatering (**Figure 10**). In addition, LOC112003582 and LOC11994737 showed changes in *Q. acutissima*, whereas LOC112040181 showed changes only in *Q. palustris*. Their expression decreased and then recovered, which is consistent with our results for IAA levels. They are involved in cell elongation and growth, and their expression is generally decreased under stress; however, these have not yet been well identified (Stortenbeker and Bemer, 2019). PYL is an ABA receptor, and the PYR/PYL/RCAR receptors that recognize ABA inhibit type 2C protein phosphatases (PP2Cs) and activate (SNF1)-related protein kinases 2 (SnRK2s) to transmit ABA response signals (Fidler et al., 2022). The expression of these genes (LOC112017749 and LOC11988217) decreased during drought, but the ABA levels were increased. This could be the result of feedback that regulates ABA responses. TIFY (TIF(F/Y)XG) proteins are key regulators of the jasmonic acid signaling pathway; they participate in defense and stress responses during plant development, and play important roles in responses to biotic and abiotic stresses (Wang et al., 2023). LOC112037230, LOC112025728, LOC112035090, LOC112035091, and LOC112015508 are TIFY protein-related genes, and their expression was changed only in *Q. palustris*. The xyloglucan endotransglucosylase/hydrolase (XTH) family plays important roles in plant cell-wall reorganization, shape maintenance, and stress resistance (Cheng et al., 2021). Xyloglucan endotransglucosylase/hydrolase-related DEGs identified only in *Q. palustris* were LOC112028320, LOC112028317, LOC112028409, LOC112022146, and LOC112013949, the levels of which tended to increase during drought and to decrease upon rewatering. Expression changes were confirmed in both two species with similar trends for LOC112028322 and LOC112028408. However, a different response was observed only for LOC112028319, the levels of which increased at the beginning of rewatering in the D vs. R1 comparison. When IAA-amido-synthase-related genes were resupplied in *Q. acutissima*, levels of LOC112008862 and LOC112013268 decreased or increased, respectively. In *Q. palustris*, levels of LOC112008862 and LOC112024715 increased and recovered, and those of LOC112013268 decreased in the D vs. R6 and C vs. R6 comparisons. On the contrary, in *Q. acutissima*, LOC112008862 expression decreased in the C vs. R6 comparison, and LOC112013268 expression increased in the D vs. R6 comparison. The results of transcriptome and concentration analysis also showed differences in sensitivity to IAA regulation between the two oaks. IAA-amido-synthase maintains auxin homeostasis by conjugating excess IAA to amino acids (Ding et al., 2008). In addition, many DEGs related to auxin-responsive and auxin-induced proteins were observed and showed various patterns depending on the treatment group. These changes in gene expression may have influenced the changes in IAA levels following drought and recovery.

### 4.6 Overview of the response of the two oak species to drought and rewatering

Significant differences in the DEGs were noted for *Q. acutissima* and *Q. palustris* in each treatment group. First, a much larger number of DEGs were identified in *Q. palustris* than in *Q. acutissima*. Only less than 10% of DEGs were common in the two species (**Figures 5C–F**). There were 862 and 3947 DEGs in *Q. acutissima* and *Q. palustris*, respectively, under drought stress, but these numbers were reduced to 231 (26.80% of 862 DEGs) and 327 (8.28% of 3947 DEGs), respectively, after recovery. The expression of these genes returned to normal (**Figures 5A, B**). In addition, *Q. palustris* had a greater number of drought stress-related genes. The DEGs related to recovery that appeared after rewatering were 1076 (55.4% of the total DEGs) in *Q. acutissima* and 1587 (28.7% of the total DEGs) in *Q. palustris*. However, the rate of induction of genes related to recovery was much higher in *Q. acutissima* than in *Q. palustris*, indicating that *Q. acutissima* is less stressed by drought and is more active in recovery. Among the DEGs related to recovery, 619 for *Q. acutissima* and 1302 for *Q. palustris* recovered to normal levels on the 6th day of rewatering, and these genes are presumed to be required initially for recovery from drought stress. Among the DEGs that were newly expressed after rewatering, the remaining 139 in *Q. acutissima* and 130 in *Q. palustris* were still identified in R6, and continued expression or repression of these genes is presumed to be required for recovery from drought stress. The two species also showed differences in the tendency of DEGs for recovery. In *Q. acutissima*, 337 (17.4%) DEGs in the D vs. R1 comparison and 198 (10.2%) DEGs in the D vs. R6 comparison showed a more active response immediately after rewatering. On the contrary, in *Q. palustris*, 402 (7.3%) and 707 (12.8%) DEGs were more active on the 6th day of water resupply, showing a relatively delayed recovery response. *In addition, 318 DEGs (16.4% of the total DEGs) in* Q. acutissima *and 155 DEGs (2.8% of the total DEGs) in* Q. palustris *in the C vs. R6 comparison were genes whose expression changed regardless of drought or rewatering when comparing the control and treatment groups on day 6.* It can be assumed that these DEGs are responsible for the memory response of the two oak species to recover normal growth upon rewatering. Similar results were reported for various plants (Li et al., 2019a, Li et al, 2021). These results suggest that more DEGs were identified in *Q. palustris* than in *Q. acutissima*, but a much smaller proportion of recovery-related genes was regulated.

With regard to the common significantly up- or downregulated genes in the two species, the following genes were upregulated in the C vs. D comparison: thaumatin-like protein 1 (TLP), which is involved in host defense and developmental processes (He et al., 2021); linoleate 13S-lipoxygenase 2-1, a chloroplast-like isoform involved in growth regulation; early light-induced protein 1, chloroplastic-like (ELIPs), known to protect photosynthetic organs against stress (Hutin et al., 2003); and Protein MOTHER of FT1 and TFL1 (MFT), involved in seed development, germination, and flowering time regulation (Xi et al., 2010) (**Supplementary Table 2**). Downregulated genes included galactinol synthase 2-like (GolS), which is involved in osmoprotectant synthesis, and late embryogenesis abundant protein 2-like (LEA), which plays a role in preventing the damage caused by drought stress (Chen et al., 2019). Additionally, heavy metal-associated isoprenylated plant protein 27-like (HIPP), a metallo chaperone, was identified (de Abreu-Neto et al., 2013). DEGs that continued to have an effect after rewatering were upregulated in the C vs. R6 comparison, including class I or IV heat shock proteins, which are representative stress response proteins, and acid phosphatase 1-like, which is involved in Pi regulation (Park and Seo, 2015). Additionally, hormone-related genes, such as auxin-induced protein 15A-like and ethylene-responsive transcription factor WIN-like, remained decreased. Pathogenesis-related protein 1-like, LRR receptor-like serine/threonine-protein kinase EFR, and glucan endo-1,3-beta-glucosidase-like were also decreased, indicating the possibility of differential disease resistance of the two species (Kebede and Kebede, 2021). These results show that both tree species’ growth and development were affected in response to drought stress, and that they were affected by abiotic as well as biotic stress responses, and that the effects were still evident even after significant recovery.

## 5 Conclusion

A comprehensive analysis of the phenotypic, physiological, and molecular parameters enhances our understanding of the complex mechanisms underlying drought adaptation in these oak species.

*Q. acutissima* showed an appropriate response despite delayed growth and minimal antioxidant enzyme activity in response to drought. Additionally, photosynthesis-related elements functioned without damage and consistently showed excellent overall tolerance and resilience against drought. *Q. palustris* showed adaptability but suffered severe phenotypic impairment and delayed recovery of several indices and poor drought resilience. Transcriptome analysis revealed that the number of genes involved in drought stress recovery was higher in *Q. acutissima*. Genes whose expression was altered under drought stress, showed recovery of expression after rewatering and normal levels were achieved for most of them. The DEGs related to sucrose and starch synthesis, glycolysis, antioxidant enzymes, and hormone synthesis were significantly more abundant in *Q. palustris*. DEGs that responded to drought in both species were primarily related to development, germination, growth regulation, photosynthetic organ protection, and flowering time regulation. Genes whose expression remained changed even after rewatering were mainly related to heat shock proteins, hormones, and pathogens. This study provides valuable insights into the differential responses of *Q. acutissima* and *Q. palustris* to drought stress and recovery. The results of this study provide useful information for the early establishment of seedlings for sustainable afforestation and ecosystem resilience.

## Data availability statement

The datasets presented in this study can be found in online repositories. The names of the repository/repositories and accession number(s) can be found in the article/**Supplementary Material.**

## Author contributions

T-LK: Conceptualization, Data curation, Formal Analysis, Investigation, Methodology, Validation, Visualization, Writing – original draft, Writing – review & editing. CO: Data curation, Funding acquisition, Resources, Formal Analysis, Writing – original draft. MD: Formal Analysis, Software, Visualization, Writing – original draft. SN: Data curation, Software, Visualization, Writing – original draft. KL: Conceptualization, Data curation, Funding acquisition, Project administration, Resources, Writing – review & editing. HL: Conceptualization, Data curation, Funding acquisition, Project administration, Resources, Supervision, Writing – review & editing.
