# Supplementary material for: Transcriptomic and physiological responses of Quercus acutissima and Quercus palustris to drought stress and rewatering
Source: Front Plant Sci. 2024 Aug 6;15:1430485. doi: 10.3389/fpls.2024.1430485 (PMC11333329; doi:10.3389/fpls.2024.1430485)
Supplement: Supplementary file 1 [file DataSheet_1.docx]

Supplementary Material

# Supplementary Tables

Supplementary Table 1. Sequence of primers used for qPCR in of *Quercus acutissima* and *Quercus palustris.*

| **Gene** | **Forward primer (5’→3’)** | **Reverse primer (5’→3’)** |
| --- | --- | --- |
| *LOC112031072* | TCTCACACGCTCCAGAACAC | GGATGGGACCAGAAACTCCG |
| *LOC112034378* | AGGATGCGGTGTTCATGACC | CGCAAACCCTCTCCCTTCAT |
| *LOC112037299* | GAACCAGTGGATGCCCTAGG | CCCATTTTCTGCTGCCAACC |
| *LOC112003295* | CAGACACAGCTTCCGGAGT | TTCCATCCGCTTCAACCACA |
| *LOC111986970* | GGGGAGCTCATTACCATCGG | AGAATGCAAGTAGGCCCACC |
| *LOC112028705* | GGCACTACATGGTCCGTTCA | TATGGTGTGCCCTCCTGAGA |
| *LOC111984042* | TGGATTCCGGGGAGATGAGT | GTTCAGCTCTCACCGCATCT |
| *LOC112033302* | ACCTCCCCTCTCTAACACCC | GTCATCTTGCAGCTCCTCCA |
| *LOC112001970* | GAGGAGTCGGTGAAAGTGGG | CTACGCCTCTTCAAGCCCAA |
| *LOC112005255* | CCCGTTGGATGGATCGTCAA | AGCGAACCCATTGACACCAT |
| *LOC112020169* | GAATCTAAGCGGGGCCACAT | TGCAAAAGCTGTTCCATGGC |
| *LOC112020346* | GGTGACATAGTTTGGGGCCA | GAGGAGGGGATCAGACAGGT |
| *LOC111997072* | CCGTCGATGTGCAAAAGCAA | CACAGCACCGTTCCCTACTT |
| *LOC112037230* | TGGCTCCCTCAAACGTTCAA | TTCCCTTGCCTTCTCAGCTG |
| *Actin* | GCTGGTCGTGATCTAACTG | CTTTGCAGTCTCCAACTCCT |
| *CAC* | TCTGGGAGAAGAGTGGCTACA | GAGCCACCATTCAAATCCT |

Supplementary Table 2. Summary of Sequencing information and mapping quality information.

| **Sample Name** | **Read count** | **Data in GB** | **Chemistry** | **Barcode used** | **Adapter Trimmed count** | **Read loss after trimming** | **Data loss in %** | **Alignment with Reference Genome (*Quercus lobata*)** | **Alignment with Reference Genome (*Quercus suber*)** | **Alignment with Reference Genome (*Quercus robur*)** |
| --- | --- | --- | --- | --- | --- | --- | --- | --- | --- | --- |
| QaC32-1_S171_L003 | 29095646 | 5.877320492 | 101 X 2 | AACGTTCC+AGTACTCC | 27040876 | 2054770 | 7.062121941 | 77.68% | 90.44% | 76.85% |
| QaC32-2_S172_L003 | 29823362 | 6.024319124 | 101 X 2 | GCAGAATT+TGGCCGGT | 27771793 | 2051569 | 6.87906682 | 76.39% | 89.34% | 75.63% |
| QaC32-3_S173_L003 | 26035509 | 5.259172818 | 101 X 2 | ATGAGGCC+CAATTAAC | 23912643 | 2122866 | 8.153733426 | 77.57% | 89.96% | 76.55% |
| QaD32-1_S168_L003 | 33805697 | 6.828750794 | 101 X 2 | ATGTAAGT+CATAGAGT | 31642741 | 2162956 | 6.398199688 | 77.75% | 90.88% | 77.45% |
| QaD32-2_S169_L003 | 26825830 | 5.41881766 | 101 X 2 | GCACGGAC+TGCGAGAC | 25192100 | 1633730 | 6.090137752 | 77.00% | 90.33% | 76.46% |
| QaD32-3_S170_L003 | 39226527 | 7.923758454 | 101 X 2 | GGTACCTT+GACGTCTT | 36228480 | 2998047 | 7.642907056 | 78.44% | 91.00% | 77.60% |
| QaR1-1_S174_L003 | 33544977 | 6.776085354 | 101 X 2 | ACTAAGAT+CCGCGGTT | 31560855 | 1984122 | 5.914811031 | 76.26% | 89.94% | 75.89% |
| QaR1-2_S175_L003 | 25173634 | 5.085074068 | 101 X 2 | GTCGGAGC+TTATAACC | 23345672 | 1827962 | 7.26141486 | 76.11% | 89.68% | 75.49% |
| QaR1-3_S176_L003 | 31555361 | 6.374182922 | 101 X 2 | CTTGGTAT+GGACTTGG | 29135870 | 2419491 | 7.66744833 | 76.52% | 89.96% | 75.84% |
| QaR6-1_S177_L003 | 32245488 | 6.513588576 | 101 X 2 | TCCAACGC+AAGTCCAA | 30187286 | 2058202 | 6.38291472 | 77.36% | 90.41% | 76.63% |
| QaR6-2_S178_L003 | 25492748 | 5.149535096 | 101 X 2 | CCGTGAAG+ATCCACTG | 23754581 | 1738167 | 6.818280242 | 78.30% | 91.06% | 77.58% |
| QaR6-3_S179_L003 | 28381915 | 5.73314683 | 101 X 2 | TTACAGGA+GCTTGTCA | 26482313 | 1899602 | 6.693001512 | 78.09% | 90.86% | 77.16% |
| QpC32-1_S186_L003 | 31302637 | 6.323132674 | 101 X 2 | TCGTAGTG+CCAAGTCT | 29203159 | 2099478 | 6.707032382 | 77.90% | 75.53% | 77.32% |
| QpC32-2_S187_L003 | 33014218 | 6.668872036 | 101 X 2 | CTACGACA+TTGGACTC | 30582042 | 2432176 | 7.367056218 | 77.48% | 75.15% | 77.25% |
| QpC32-3_S188_L003 | 32001345 | 6.46427169 | 101 X 2 | TAAGTGGT+GGCTTAAG | 29945213 | 2056132 | 6.425142443 | 78.37% | 76.14% | 77.95% |
| QpD32-1_S183_L003 | 28355597 | 5.727830594 | 101 X 2 | CGTTAGAA+GACCTGAA | 26446315 | 1909282 | 6.733351444 | 76.56% | 74.46% | 76.55% |
| QpD32-2_S184_L003 | 31655317 | 6.394374034 | 101 X 2 | AGCCTCAT+TCTCTACT | 29321425 | 2333892 | 7.372827762 | 77.79% | 75.65% | 77.22% |
| QpD32-3_S185_L003 | 27474607 | 5.549870614 | 101 X 2 | GATTCTGC+CTCTCGTC | 25427956 | 2046651 | 7.449245771 | 78.27% | 75.37% | 76.26% |
| QpR1-1_S189_L003 | 25839924 | 5.219664648 | 101 X 2 | CGGACAAC+AATCCGGA | 23857916 | 1982008 | 7.670332157 | 77.99% | 74.62% | 76.93% |
| QpR1-2_S190_L003 | 26520146 | 5.357069492 | 101 X 2 | ATATGGAT+TAATACAG | 23913738 | 2606408 | 9.828030359 | 77.07% | 74.02% | 75.74% |
| QpR1-3_S191_L003 | 31061463 | 6.274415526 | 101 X 2 | GCGCAAGC+CGGCGTGA | 28109050 | 2952413 | 9.505067421 | 75.97% | 73.30% | 75.74% |
| QpR6-1_S192_L003 | 31194726 | 6.301334652 | 101 X 2 | AAGATACT+ATGTAAGT | 28976599 | 2218127 | 7.110583372 | 77.60% | 75.11% | 77.20% |
| QpR6-2_S193_L003 | 31821806 | 6.428004812 | 101 X 2 | GGAGCGTC+GCACGGAC | 29766254 | 2055552 | 6.459570522 | 78.05% | 75.64% | 77.54% |
| QpR6-3_S194_L003 | 31489810 | 6.36094162 | 101 X 2 | ATGGCATG+GGTACCTT | 28546719 | 2943091 | 9.346169443 | 78.09% | 75.56% | 77.64% |

Supplementary Table 3. List of DEGs that were most commonly up- or down-regulated by both tree species in the C vs D and C vs R6 treatment groups.

|  | most upregulation of DEGs | | most downregulation of DEGs | |
| --- | --- | --- | --- | --- |
|  | Gene IDs | function | Gene IDs | function |
| C vs D | LOC112038887, LOC112038888, LOC112038876, LOC112038861, LOC112038862, LOC111989390 | thaumatin-like protein 1 | LOC112007268 | galactinol synthase 2-like |
|  | LOC111990529 | linoleate 13S-lipoxygenase 2-1, chloroplast-like isoform X1 | LOC112029982 | cyclin-SDS |
|  | LOC112031465 | uncharacterized protein | LOC111983933 | late embryogenesis abundant protein 2-like |
|  | LOC111995089 | uncharacterized protein | LOC112032815 | membrane protein PM19L |
|  | LOC112021193 | uncharacterized protein | LOC112008221 | heavy metal-associated isoprenylated plant protein 27-like |
|  | LOC112032815 | membrane protein PM19L | LOC111983007 | RNA-binding motif protein, X-linked-like-3 isoform X3 |
|  | LOC112025257 | cysteine proteinase inhibitor B-like, partial | LOC112018691 | dehydrin Rab18-like |
|  | LOC111987464 | early light-induced protein 1, chloroplastic-like | LOC111990835 | late embryogenesis abundant protein 2-like |
|  | LOC112038587 | protein MOTHER of FT and TFL1 | LOC111984751 | cucumber peeling cupredoxin-like |
|  | LOC111984751 | cucumber peeling cupredoxin-like | LOC111997339 | uncharacterized protein |
|  | LOC111984042 | probable protein phosphatase 2C 51 | LOC111983863 | RNA-binding motif protein, X-linked-like-3 |
|  |  |  | LOC112023339 | galactinol synthase 2-like, partial |
|  |  |  | LOC111984042 | probable protein phosphatase 2C 51 |
| C vs R6 | LOC112008844 | acid phosphatase 1-like | LOC112029728 | germin-like protein subfamily 1 member 16 |
|  | LOC111990377 | 22.0 kDa class IV heat shock protein-like | LOC111994734 | auxin-induced protein 15A-like |
|  | LOC112008845 | acid phosphatase 1-like | LOC112021260 | LRR receptor-like serine/threonine-protein kinase EFR, partial |
|  | LOC112016015 | acid phosphatase 1-like | LOC112016185 | pathogenesis-related protein 1-like |
|  | LOC111983939 | probable terpene synthase 9 | LOC112008483 | wall-associated receptor kinase 2-like |
|  | LOC112017482 | 17.5 kDa class I heat shock protein-like | LOC112013165 | phylloplanin-like |
|  | LOC112016014 | acid phosphatase 1-like | LOC112033155 | glu *S.griseus* protease inhibitor-like |
|  | LOC111993015 | flavonoid ’\\'-monooxygenase-like | LOC112041147 | glucan endo-1,3-beta-glucosidase-like |
|  | LOC112024436 | 17.5 kDa class I heat shock protein-like | LOC112015556 | EG45-like domain containing protein |
|  | LOC112016302 | 22.0 kDa class IV heat shock protein-like | LOC112002896 | ethylene-responsive transcription factor WIN1-like |

Supplementary Figure 1**.** Correlation of RNA-Seq Data from Different Libraries. Scatterplot comparison of biological replicates based on A) samples and B) genes-based, PC1, first principle component; PC2, second principle component. R, Pearson correlation coefficient; C) Heatmap showing the principal component analysis (PCA) of expression in R cells for four time points; (D) Correlograms showing the correlation score matrix across all the pairwise libraries at all time points.


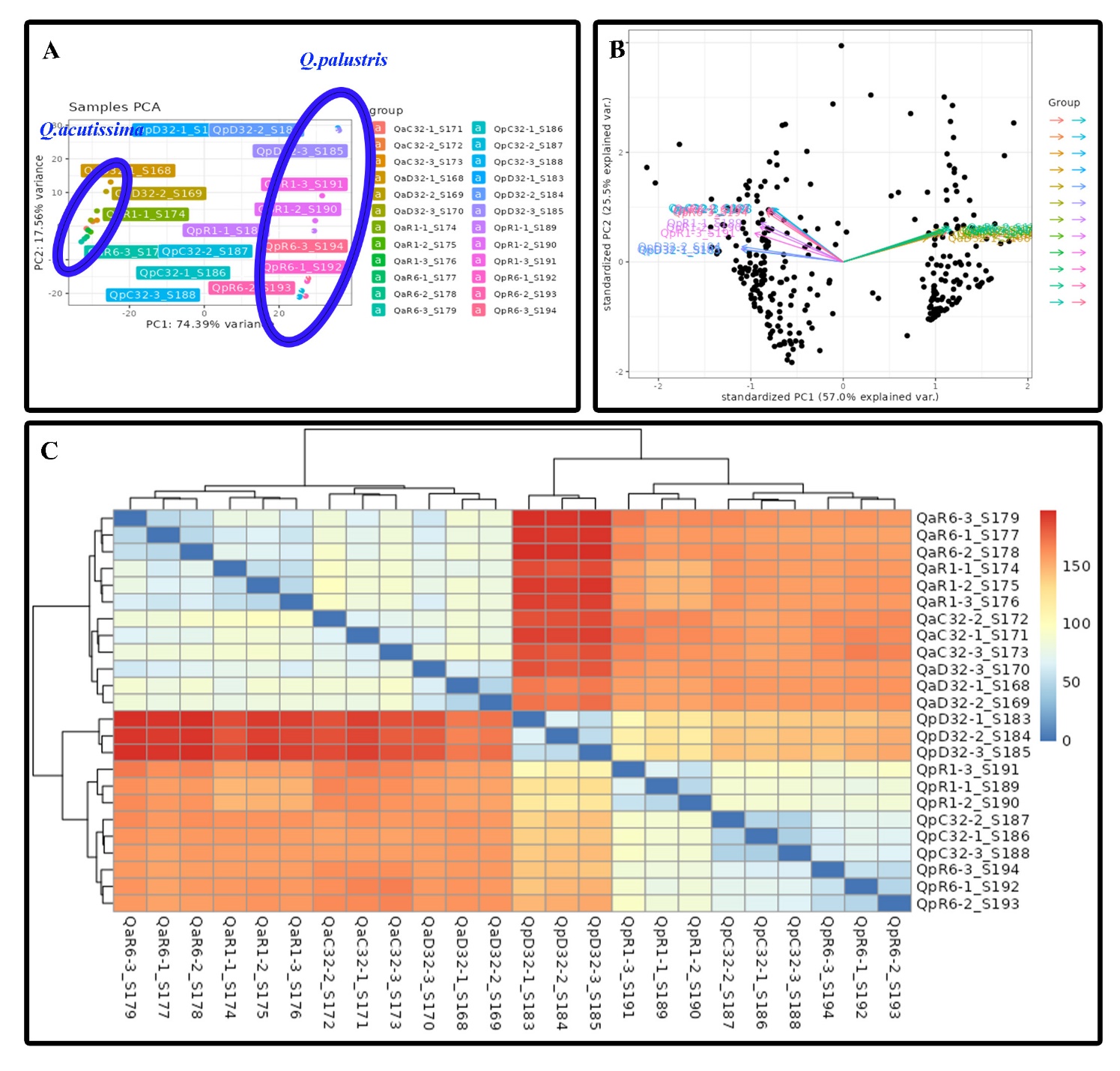


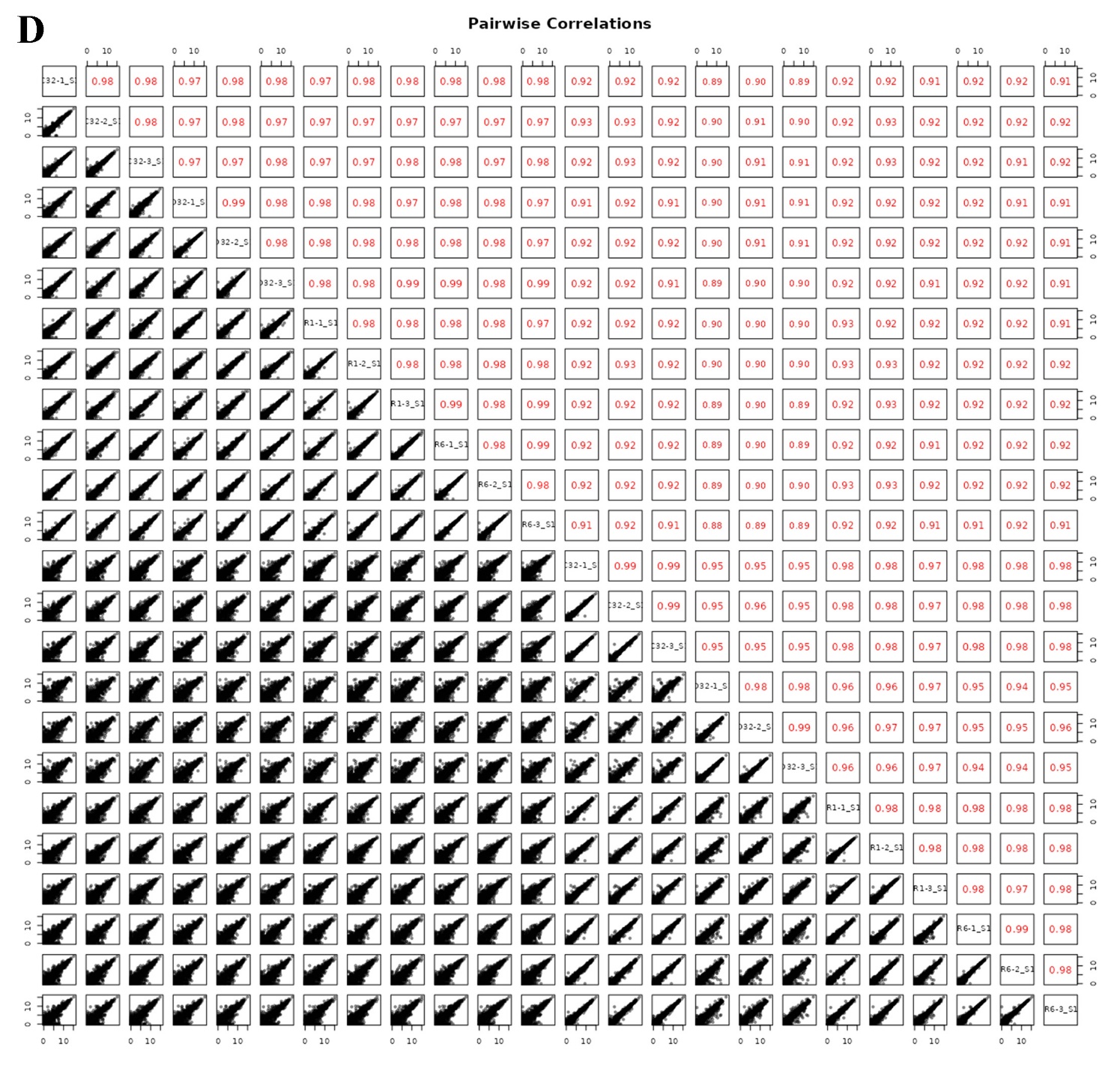


Supplementary Figure 2 : Functional analysis of DEGs and comparison of the enrichment scores of upregulated and downregulated DEGs in *Q. acutissima* and *Q. palustris*. Bubble graph for GO enrichment.


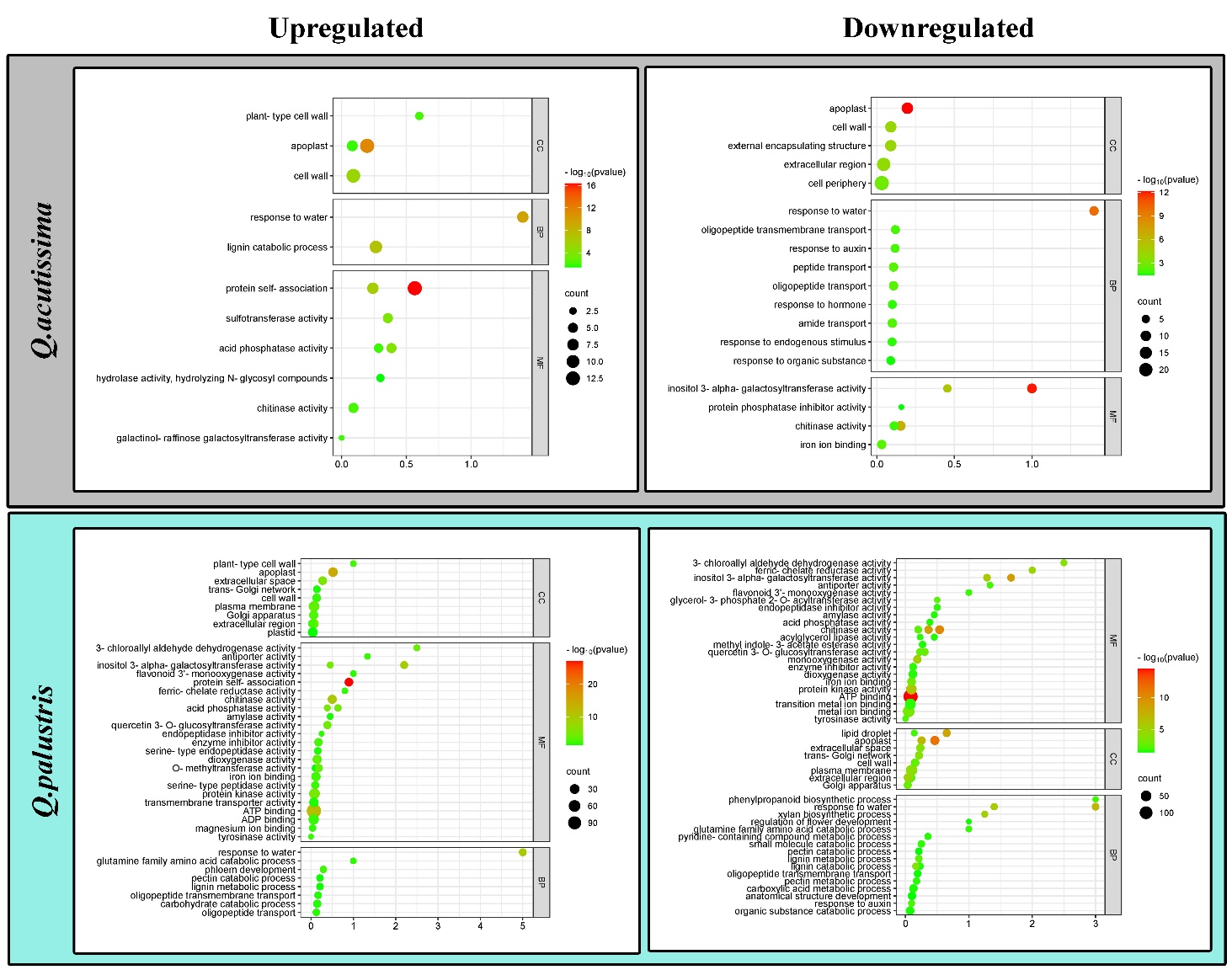


Supplementary Figure 3. Line graph showing number of differentially expressed genes (DEGs) related to the antioxidant pathways under different experiment conditions (C vs. D, D vs. R1, D vs. R6, and C vs. R6) across *Q. acutissima* (black) and *Q. palustris* (white).


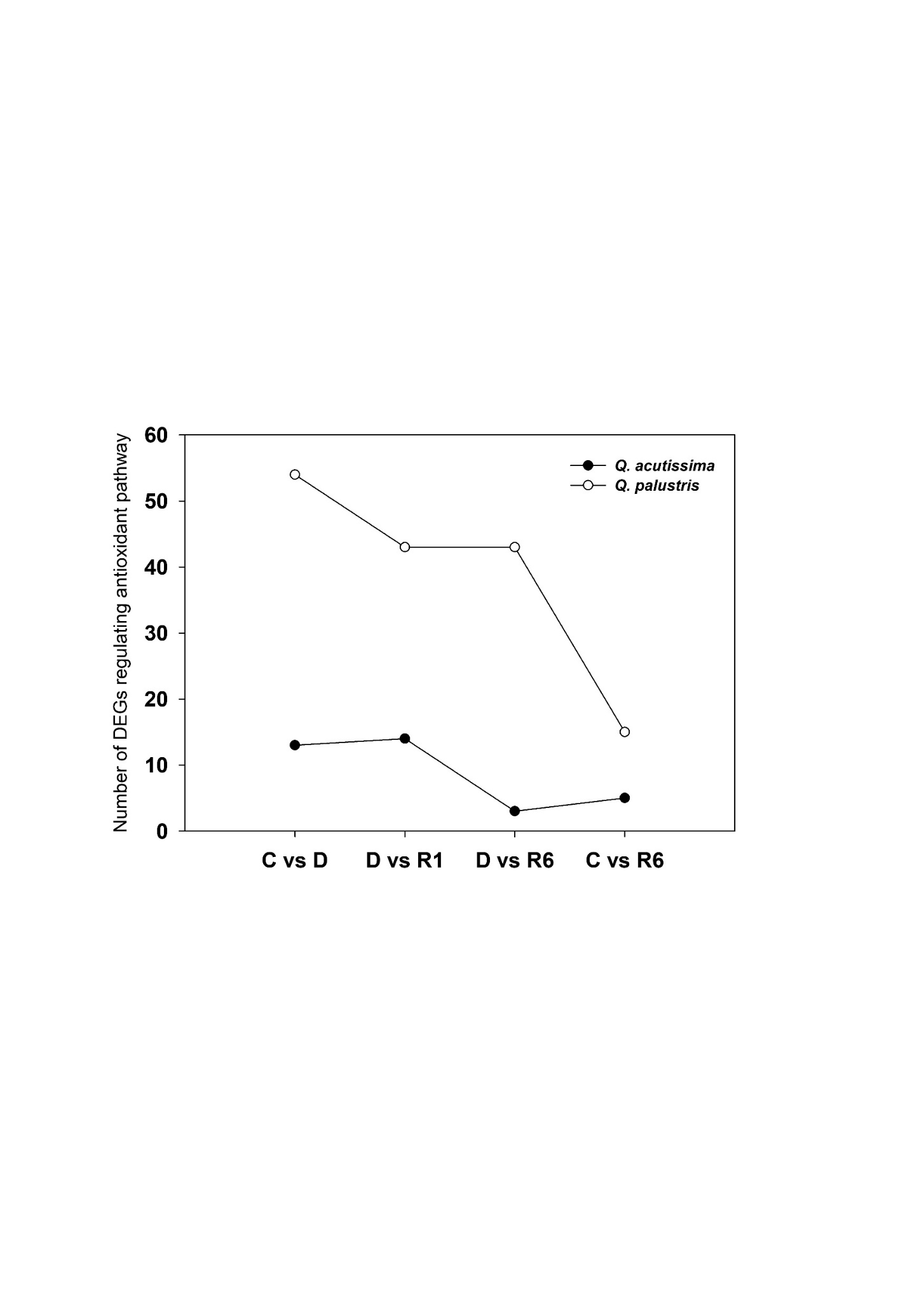


13

14

3

5

54

43

43

15

Supplementary Figure 4. Linear regression analysis of fold change of the gene expression between RNA-SEQ and qPCR.
